# NNMT/1‐MNA Promote Cell‐Cycle Progression of Breast Cancer by Targeting UBC12/Cullin‐1‐Mediated Degradation of P27 Proteins

**DOI:** 10.1002/advs.202305907

**Published:** 2023-12-21

**Authors:** Yilei Ma, Xucheng Huang, Yanzhong Wang, Yinjiao Lei, Jinwei Yu, Shaobo Yu, Yuzhen Gao, Jun Yang, Feng Zhao, Haitao Yu, Jin Zeng, Yadong Chu, Min Yang, Guoli Li, Xinyou Xie, Jun Zhang

**Affiliations:** ^1^ Department of Clinical Laboratory Sir Run Run Shaw Hospital School of Medicine Zhejiang University Hangzhou Zhejiang 310016 P. R. China; ^2^ Key Laboratory of Precision Medicine in Diagnosis and Monitoring Research of Zhejiang Province Hangzhou Zhejiang 310016 P. R. China; ^3^ Department of Pathology Sir Run Run Shaw Hospital School of Medicine Zhejiang University Hangzhou Zhejiang 310016 P. R. China; ^4^ Department of Cytopathology Ningbo Diagnostic Pathology Center Ningbo Zhejiang 315046 P. R. China; ^5^ Department of Clinical Laboratory Zhejiang Armed Police Corps Hospital Hangzhou Zhejiang 310051 P. R. China

**Keywords:** breast cancer, cell‐cycle, neddylation, NNMT/1‐MNA, p27, UBC12

## Abstract

Cell cycle dysregulation is a defining feature of breast cancer. Here, 1‐methyl‐nicotinamide (1‐MNA), metabolite of nicotinamide N‐methyltransferase(NNMT) is identified, as a novel driver of cell‐cycle progression in breast cancer. NNMT, highly expressed in breast cancer tissues, positively correlates with tumor grade, TNM stage, Ki‐67 index, and tumor size. Ablation of NNMT expression dramatically suppresses cell proliferation and causes cell‐cycle arrest in G0/G1 phase. This phenomenon predominantly stems from the targeted action of 1‐MNA, resulting in a specific down‐regulation of p27 protein expression. Mechanistically, 1‐MNA expedites the degradation of p27 proteins by enhancing cullin‐1 neddylation, crucial for the activation of Cullin‐1‐RING E3 ubiquitin ligase(CRL1)—an E3 ubiquitin ligase targeting p27 proteins.  NNMT/1‐MNA specifically up‐regulates the expression of UBC12, an E2 NEDD8‐conjugating enzyme required for cullin‐1 neddylation. 1‐MNA showes high binding affinity to UBC12, extending the half‐life of UBC12 proteins via preventing their localization to lysosome for degradation. Therefore, 1‐MNA is a bioactive metabolite that promotes breast cancer progression by reinforcing neddylation pathway‐mediated p27 degradation. The study unveils the link between NNMT enzymatic activity with cell‐cycle progression, indicating that 1‐MNA may be involved in the remodeling of tumor microenvironment.

## Graphical abstract

In this study, it is demonstrated that the NNMT‐specific metabolite, 1‐methyl‐nicotinamide (1‐MNA), promotes breast cancer cell‐cycle progression by enhancing CRL1‐mediated degradation of p27 proteins. This is facilitated by 1‐MNA's direct interaction with UBC12 proteins, preventing their lysosomal degradation and increasing cullin‐1 neddylation, crucial for CRL1 ligase activation. The findings suggest NNMT/1‐MNA's potential role in reshaping the tumor microenvironment.

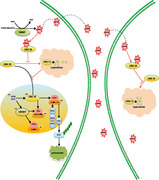

## 1 Introduction

Breast cancer continues to be the most frequently diagnosed cancer among women worldwide and ranks as the second leading cause of cancer‐related deaths in developed countries, following lung cancer.^[^ ^1^, ^2^ ^]^ Dysregulation of the cell cycle, characterized by the inactivation of tumor suppressors and abnormal activation of cyclins and cyclin‐dependent kinases (CDKs), is a defining feature of breast cancer.^[^ ^3^, ^4^ ^]^ It manifests as uncontrolled cell proliferation and aberrant cell cycle progression in cancer cells.^[^ ^5^ ^]^ Agents targeting cell‐cycle regulators, such as palbociclib, ribociclib, and abemaciclib, have been approved for breast cancer treatment.^[^ ^6^ ^]^ However, despite this significant progress, disease heterogeneity, lack of target specificity and dose‐limiting toxicities continue to impose significant clinical obstacles that restrict the overall survival of breast cancer patients.^[^ ^3^ ^]^ Therefore, gaining a comprehensive understanding of cell‐cycle progression and its regulatory mechanisms not only illuminates the intricate biology of breast cancer but also unveils potential therapeutic targets.

Metabolic change is closely linked to cell‐cycle progression, as both processes require coordination of various signals and pathways to ensure the production of sufficient biomass and energy for cell division.^[^ ^7^, ^8^ ^]^ For example, oncogene MYC can induce the expression of genes involved in glycolysis, glutaminolysis, nucleotide biosynthesis, and fatty acid synthesis, as well as promote cell cycle entry by activating CDKs and inhibiting CDK inhibitors (CKIs).^[^ ^9^, ^10^ ^]^ On the other hand, metabolic process can also affect cell‐cycle progression by altering the levels of certain metabolites that act as signaling molecules or cofactors for cell cycle regulators. For instance, acetyl‐CoA can serve as a substrate for histone acetyltransferases (HATs), which can modulate the expression of genes involved in cell cycle control.^[^ ^11^ ^]^ Similarly, NAD+ can modulate the activity of sirtuins, which are histone deacetylases (HDACs) that can affect cell cycle checkpoints.^[^ ^12^, ^13^ ^]^ There is significant value in characterizing metabolic pathways and metabolites that contribute to the maximized growth and proliferation of cancer cells in breast cancer.

Nicotinamide N‐methyltransferase (NNMT) is a cytosolic enzyme which catalyzes transferring of a methyl group from S‐adenosyl‐L‐methionine (SAM) to nicotinamide (NA), resulting in the formation of 1‐methyl‐nicotinamide (1‐MNA) and S‐adenosyl‐L‐homocysteine (SAH).^[^ ^14^ ^]^ Mainly expressed in liver tissue, NNMT functions to regulate histone methylation, polyamine flux and NAD‐dependent SIRT1 signaling.^[^ ^15^ ^]^ In recent decades, NNMT has been found to be highly expressed in multiple human cancers, including breast cancer.^[^ ^16^ ^]^ Through depletion of SAM and reduction in histone methylation, the expression of NNMT in stromal cells promoted migration, proliferation, in vivo growth, and metastasis of ovarian cancer.^[^ ^14^ ^]^ Our own researches demonstrated that NNMT contributes to high metastasis of TNBC by impacting membrane fluidity and enhance chemoresistance of breast cancer by promoting Sirt1 protein stabilization.^[^ ^17^, ^18^ ^]^ Besides these, NNMT has been shown to play a positive regulatory role in cell‐cycle progression and cell proliferation in human colorectal cancer, though the underlying mechanisms have not been fully determined.^[^ ^19^ ^]^ More interestingly, Hong et al. reported that 1‐MNA can stabilize SIRT1 by decreasing its ubiquitination.^[^ ^20^ ^]^ One recent study found that 1‐MNA could induce T cells to secrete the tumor‐promoting cytokine tumor necrosis factor α (TNFα) in human ovarian cancer.^[^ ^21^ ^]^ All these studies substantiated cancer‐promoting role of NNMT and the biological activity of its metabolite, 1‐MNA. However, in breast cancer, the precise biological role of NNMT and its metabolite 1‐MNA in cell‐cycle progression, as well as the underlying mechanisms, remain largely unknown and undisclosed.

Neddylation is a post‐translation modification (PTM) that conjugates of ubiquitin‐like molecule NEDD8 to substrate proteins with unique sequential enzymatic reactions of activating enzyme(E1), conjugating enzyme(E2), and ligase(E3).^[^ ^22^, ^23^ ^]^ Up to now, one heterodimer NEDD8 E1‐activating enzyme (APPBP1–UBA3), two kinds of NEDD8 E2‐conjugating enzymes (UBE2F, and UBC12, also known as UBE2M), and many kinds of E3 ligases have been found to act in concert in neddylation.^[^ ^23^ ^]^ The well‐established substrates of neddylation are the cullin subunits (Cullin1, 2, 3, 4 and 5) of cullin‐RING ligases (CRLs).^[^ ^23^ ^]^ CRLs represent the largest family of multiunit E3 ubiquitin ligases and play a crucial role in the degradation of ≈20% of proteasome‐regulated proteins, involving in many aspects of important biological processes, including cell‐cycle and cell proliferation.^[^ ^23^ ^]^ Aberrant activation of neddylation is commonly observed in human cancers, and targeting neddylation has emerged as a potential therapeutic strategy for cancer treatment.^[^ ^24^ ^]^

In this research, we found that NNMT expression in breast cancer tissue showed positive correlation with tumor size, histological grade, and tumor cell proliferation (Ki‐67 index), and high level of NNMT predicts poor survival. In‐vitro study verify that NNMT and its metabolite 1‐MNA promote proliferation and cell‐cycle progression of breast cancer cells by down‐regulating p27 expression. Mechanistically, 1‐MNA facilitates degradation of p27 proteins by increasing cullin‐1 neddylation, which is prerequisite for CRL1 ligase activation. Further studies demonstrated that 1‐MNA directly interacts with UBC12 proteins, and the neddylation‐promoting effect by 1‐MNA is due to its ability to specifically protect UBC12 from localization to lysosome for degradation. Collectively, our study identified UBC12 as a intracellular target protein of 1‐MNA and demonstrated that NNMT/1‐MNA pathway promote breast cancer progression through engaging neddylation‐mediated degradation of p27 proteins.

## 2 Results

### 2.1 NNMT Expression Positively Correlated with Breast Cancer Progression

**Figure 1:**
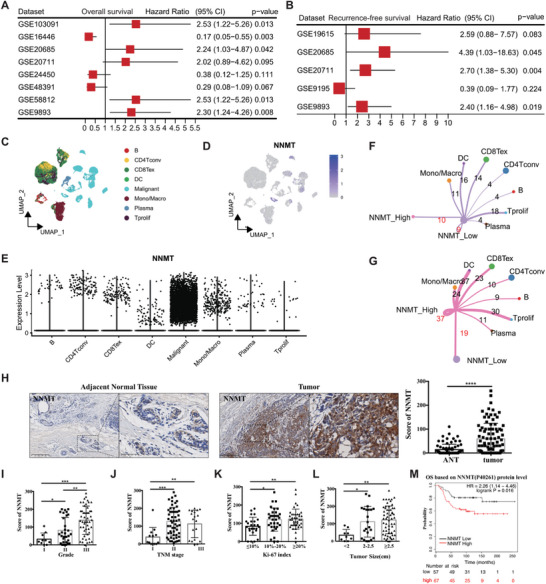
NNMT expression positively correlated with breast cancer progression. A) Cox analysis for NNMT in eight public cohorts with OS data. B) Cox analysis for NNMT in five public cohorts with RFS data. C) the umap of scRNA for main cell types in the scRNA cohort (GSE176078). D) the featureplot of NNMT expression in each cell in scRNA of breast cancer. E, NNMT is highly expressed in the breast malignant cells. Breast cancer cells were grouped into NNMT high group and NNMT low group in scRNA data. By using Cellchat R packages, the cell communications between high NNMT tumor cells and other cell types F) and the cell communications between low NNMT tumor cells and other cell types G) were identified. H) IHC(Immunohistochemistry) staining of human breast cancer tissue and adjacent normal tissue(ANT) using specific antibodies against NNMT. The expression level of NNMT in breast cancer tissue and ANT was scored from 0 to 300 according to the staining intensity. The expression pattern of NNMT was analyzed based on histological grade I), TNM stage J), Ki‐67 index K), and tumor size L). M) Overall survival(OS) analysis of breast cancer patients based on the protein level of NNMT using dataset from the program Kaplan–Meier plotter (http://kmplot.com/analysis). Data were presented as mean ± SEM. *, *P*<0.05; **, *P*<0.001; ***, *P*<0.001.

To identify the prognostic value of NNMT in breast cancer, we collected ten cohorts of breast cancer patients with bulk RNA transcripts from the public GEO repository. After conducting Cox analysis on NNMT in eight publicly available cohorts with overall survival (OS) data, our findings indicated that patients with high NNMT expression exhibited a significantly poorer OS rate compared to those with low NNMT expression in five out of the eight breast cancer cohorts (**Figure** **1**A). Moreover, a separate analysis of NNMT in five public cohorts with recurrence‐free survival (RFS) data revealed that four out of five breast cancer patient cohorts with high NNMT expression had a worse prognosis than those with low NNMT expression (Figure 1B). Furthermore, investigating the single‐cell expression of NNMT, we observed predominant expression in tumor cells, with minimal expression detected in immune cells (Figure 1C–E). Through cell communication analysis, we identified that tumor cells with high NNMT expression exerted a more substantial impact on other cells, including both tumor cells and immune cells, when compared to tumor cells with low NNMT expression (Figure 1F,G). Collectively, these analyses strongly indicated the unfavorable role of NNMT in breast cancer patients.

To verify above research and further characterize the clinicopathological features of NNMT in breast cancer, we investigated NNMT expression in breast cancer tissues and adjacent normal tissue(ANT) from 90 breast cancer patients by immunohistochemistry staining (IHC). Based on the staining intensity, the levels of NNMT expression in samples were scored from 0 to 300. The data showed that breast cancer tissues included in this study displayed significantly higher level of NNMT staining than that of ANT (Figure 1H). When examining tumor grade, which serves as an indicator of a tumor's aggressiveness and growth potential, we observed that breast cancer tissues classified as moderately differentiated (Grade II) and poorly differentiated (Grade III) expressed significantly higher levels of NNMT compared to well‐differentiated (Grade I) cancer tissues (Figure 1I). Additionally, in our investigation, we found a significant correlation between the level of NNMT expression and TNM stages in breast cancer patients, with higher stage of breast cancer showing notably higher NNMT levels (Figure 1J). Moreover, NNMT is significantly higher stained in breast cancer tissues exhibiting medium to high Ki‐67 index values, which serves as a marker reflecting the growth potential of cancer cells (Figure 1K). These findings suggest a potential association between NNMT expression and tumor growth in breast cancer. Upon evaluating the tumor size, we found that breast cancer patients with larger tumor volumes tend to express higher levels of NNMT (Figure 1L). When we plotted NNMT expression against the survival of breast cancer patients using protein datasets (including 112 patients) from the Kaplan‐Meier plotter(http://kmplot.com/analysis/),^[^ ^25^ ^]^ breast cancer patients with relatively higher levels of NNMT displayed significantly poorer overall survival (OS) (Figure 1M). Taken together, NNMT expression is elevated in breast cancer, and high level of NNMT may promote cancer progression by regulating cancer cell proliferation.

### 2.2 NNMT Promote Cell‐Cycle Progression Through Down‐Regulating p27 Expression

**Figure 2:**
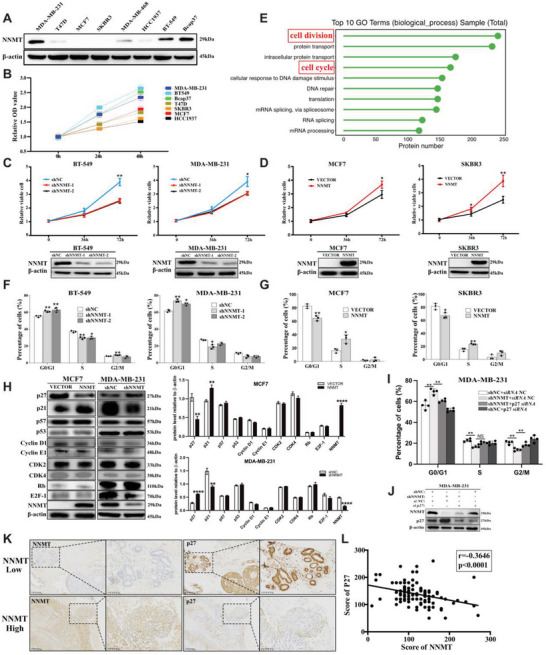
NNMT promote cell‐cycle progression through down‐regulating p27 expression. A) Western‐blot detection of NNMT in indicated distinct human breast cancer cell lines (SKBR3, MCF7, MDA‐MB‐231, BT‐549, HCC1937, Bcap37, T47D and MDA‐MB‐468). B) the cell viabilities of above distinct human breast cancer cell lines were tested by Cell‐Counting Kit‐8 (CCK8) colorimetric assay at indicated time points (0 h, 24 h and 48 h) and relative viable cells were calculated. C) the relative cell viability was analyzed by Cell‐Counting Kit‐8 (CCK8) colorimetric assay in MDA‐MB‐231 cells and BT‐549 cells transfected with two distinct NNMT specific shRNA (1# and 2#) or control shRNA. D) the relative cell viability of SKBR3 cells transfected with empty vector(SKBR3/VECTOR) or NNMT‐overexpressing plasmids(SKBR3/NNMT), and MCF7 cells transfected with empty vector(MCF7/VECTOR) or NNMT‐overexpressing plasmids (MCF7/NNMT). E) MDA‐MB‐231/NC cells and MDA‐MB‐231/shNNMT cells were subjected to protein mass spectrometry, and the top 10 biological processes were displayed upon GO (gene ontology) analysis of all the significantly differential expressed proteins. F) cell‐cycle progression was analyzed by flow cytometry in MDA‐MB‐231 cells and BT‐549 cells transfected with two distinct NNMT specific shRNA (1# and 2#) or control shRNA, results were calculated as percentage of cells in G0/G1 phase, S phase and G2/M phase. G) flow cytometry analysis of cell‐cycle progression of SKBR3 cells transfected with empty vector(SKBR3/VECTOR) or NNMT‐over‐expressing plasmids(SKBR3/NNMT), and MCF7 cells transfected with empty vector(MCF7/VECTOR) or NNMT‐over‐expressing plasmids(MCF7/NNMT), and the results were calculated as percentage of cells in G0/G1 phase, S phase and G2/M phase. H) western‐blot detection of p21, p27, p57, p53, CDK2, CDK4, Cyclin E1, Cyclin D1, Rb, E2F‐1, and NNMT in indicated cell lysates, and their protein levels relative to β‐actin were calculated based on the the grayscale values of the bands. I) flow cytometry analysis of cell‐cycle progression of MDA‐MB‐231 transfected with NNMT specific shRNA or control shRNA, and p27 siRNA or control siRNA, and the results were calculated as percentage of cells in G0/G1 phase, S phase and G2/M phase. J) western‐blot detection of p27 and NNMT in lysates of MDA‐MB‐231 cells with indicated treatments. K) IHC(Immunohistochemistry) staining of NNMT and p27 protein expression in human breast cancer tissue. L) Pearson correlation analysis of the association between NNMT and p27 protein level in breast cancer tissue (*r* = −0.3815, *P* = 0.0001). Data are representative of three independent experiments. Data were presented as mean ± SEM. *, *P*<0.05; **, *P*<0.01; ****, *P*<0.0001.

To verify the contribution of NNMT to breast cancer progression, we examined NNMT expression in eight breast cancer cell lines (SKBR3, MCF7, MDA‐MB‐231, BT‐549, HCC1937, Bcap37, T47D, and MDA‐MB‐468) by western‐blot. Among them, MDA‐MB‐231, BT‐549, and Bcap37 cells exhibit significant higher level of NNMT expression than other cell lines, whereas SKBR3 and MCF7 cells lack NNMT expression (**Figure** **2**A). When comparing the cell proliferation ability of these cell lines, we found that NNMT‐high expressing cell lines (MDA‐MB‐231, BT‐549, and Bcap37 cells) demonstrate a stronger proliferation ability compared to other NNMT‐low expressing (T47D, MDA‐MB‐468, and HCC1937 cells) and NNMT‐absent SKBR3 and MCF7 cells (Figure 2B), indicating the involvement of NNMT in breast cancer cell proliferation. To verify this, we introduced NNMT expression into MCF7 and SKBR3 cell lines, or knockdown of NNMT expression in MDA‐231 and BT‐549 by NNMT shRNA for subsequent studies. As shown in Figure 2C, reduction of NNMT expression in MDA‐MB‐231 and BT‐549 cells using two distinct NNMT specific shRNA resulted in a robust inhibition of their proliferation. Conversely, the introduction of NNMT into NNMT‐blunt breast cancer cell lines (MCF7 and SKBR3) significantly enhanced their proliferative capacity (Figure 2D). These confirmed the role of NNMT in promoting breast cancer cell proliferation. To explore the underlying biological process regulated by NNMT in breast cancer cells, we compared the proteome between control shRNA and NNMT‐shRNA transfected MDA‐MB‐231 cells by protein mass spectrometry analysis. GO (gene ontology) analysis of all the significantly differential expressed proteins (Table S3, Supporting Information) showed that high percentage of the proteins influenced by NNMT knockdown were enriched in the biological process of cell division and cell‐cycle (Figure 2E; Table S4, Supporting Information). Consistently, when NNMT expression was silenced by shRNA, cell‐cycle arrest occurred in the G0/G1 and S phase in both MDA‐MB‐231 and BT‐549 cells compared to control shRNA treated cells (Figure 2F). Conversely, overexpression of NNMT promoted cell‐cycle progression from G0/G1 phase to S phase in MCF7 and SKBR3 cells (Figure 2G). Therefore, in addition to the anti‐apoptotic capability as mentioned in our previous study,^[^ ^26^ ^]^ NNMT also possesses the ability to promote cell cycle progression in facilitating breast cancer cell proliferation.

CDK inhibitors, including p21, p27, and p57, are well‐known for their roles in regulating G1‐S and/or G2‐M transitions by inhibiting the activity of CDK‐cyclin complexes and their dysregulation are commonly observed in breast cancer.^[^ ^27^, ^28^ ^]^ In our previous colon cancer research, we observed a substantial increase in p27 expression upon silencing NNMT in HT‐29 cells.^[^ ^19^ ^]^ Therefore, we explored the impact of NNMT knockdown on cell cycle arrest in breast cancer cells by examining the expression of key cell‐cycle regulators (p21, p27, p57, p53, CDK2, CDK4, Cyclin E1, Cyclin D1, Rb, and E2F‐1) through western blot analysis, quantifying their expression levels normalized to β‐actin to gain a comprehensive understanding of the associated molecular changes. As shown in Figure 2H, the knockdown of NNMT expression in MDA‐MB‐231 cells resulted in a notable increase in p27 protein expression and a decrease of p21 proteins compared to cells treated with control shRNA, with minimal impact on other tested proteins. And vice versa, the over‐expression of NNMT in MCF7 cells led to a specific down‐regulation of p27 expression and an increase in p21 proteins (Figure 2H). Both p27 and p21 are well‐documented cyclin‐dependent kinase inhibitors that prevent uncontrolled cell growth by blocking the transition from G1 phase to S phase of the cell cycle. We hypothesize that p27, other than p21, is the downstream target of NNMT involved in promoting cell‐cycle progression in breast cancer cells. This was further supported by the observation that siRNA‐mediated silencing of p27 expression in NNMT‐silenced MDA‐MB‐231 cells (MDA‐MB‐231/shNNMT) could partially reverse the G0/G1 and G2 cell phase arrest caused by NNMT knockdown (Figure 2I,J). Next, we conducted immunohistochemistry staining of p27 in breast cancer tissues and observed that breast cancer tissues with high NNMT expression displayed lower levels of p27 staining compared to NNMT‐low breast cancer tissues (Figure 2K). Additionally, we confirmed a significant negative correlation (r = −0.3646, p<0.0001) between NNMT and p27 expression in breast cancer tissues (Figure 2L). Taken together, in breast cancer, NNMT promotes cell‐cycle progression by reducing p27 expression.

### 2.3 1‐MNA, the Metabolite of NNMT, is the Key Molecule that Promotes the Progression of Breast cancer Cell Cycle

**Figure 3:**
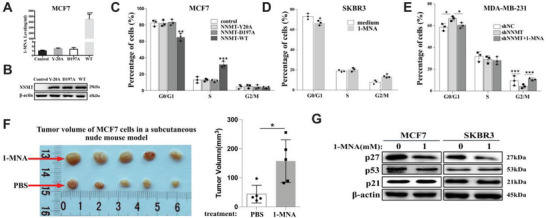
1‐MNA, the metabolite of NNMT, is the key molecule that promotes the progression of breast cancer cell cycle. A,B) Lentivirus mediated over‐expression of wide type NNMT(NNMT‐wt) or two different site‐specific mutated NNMT (NNMT‐Y20A and NNMT‐D197A) in MCF7 cell lines, then quantification of 1‐MNA in cell lysate was determined using LC‐MS/MS (A) and protein level of UBC12 and NNMT was determined by western‐blot in cell lysates (B). C) flow cytometry analysis of cell‐cycle progression of MCF7 cell transfected with lentivirus harboring NNMT‐WT, NNMT‐Y20A and NNMT‐D197A mutants, or control lentivirus, and the results were calculated as percentage of cells in G0/G1 phase, S phase and G2/M phase. D) flow cytometry analysis of cell‐cycle progression of SKBR3 cells stimulated with medium or 1‐MNA (1 mm, 24 h), and the results were calculated as percentage of cells in G0/G1 phase, S phase and G2/M phase. E) flow cytometry analysis of cell‐cycle progression of MDA‐MB‐231 cells transfected with control shRNA(shNC), shRNA targeting NNMT(shNNMT), and shNNMT plus 1‐MNA (1 mm, 24 h), and the results were calculated as percentage of cells in G0/G1 phase, S phase and G2/M phase. F) 10 days after MCF‐7 xenograft mouse model was established, the mouse were intraperitoneally injected with 1‐MNA (500 mg kg^−1^) or sterile PBS every 2 days for consecutive two weeks, and tumor volume was calculated according to V = (length × width^2^)/2. G) MCF7 and SKBR3 cells were stimulated with or without 1‐MNA(1.0 mm) for 24 h, then the protein level of p27, p53, and p21 were detected using western‐blot. Data are representative of three independent experiments. Data were presented as mean ± SEM. *, *P*<0.05; **, *P*<0.001; ***, *P*<0.001.

Then we wanted to know whether N‐methyltransferase activity is essential for its effect on cell‐cycle progression of breast cancer. Previous structural analysis of NNMT revealed two critical amino acid residues (D197 and Y20) that are essential for its N‐methyltransferase activity.^[^ ^29^ ^]^ Indeed, MCF‐7 cells transfected with either D197A‐NNMT or Y20A‐NNMT mutant displayed more than 90% loss of 1‐MNA release into the supernatant comparing to cells transfected with WT‐NNMT, even nearly equivalent amount of NNMT proteins were detected among NNMT‐WT and the mutants transfected MCF‐7 cells (**Figure** **3**A,B). However, when examining the cell‐cycle progression of MCF‐7 cells, we observed that only overexpression of NNMT‐WT, not NNMT‐Y20A or NNMT‐D197A, resulted in an increased cell‐cycle progression from G0/G1 to S phase (Figure 3C). These results indicate the essentiality of NNMT's N‐methyltransferase activity in promoting breast cancer progression. We and other groups have reported that 1‐MNA, one of the metabolites generated from N‐methyltransferase reaction of NNMT, somehow demonstrated cancer‐promoting effect and immune regulatory effect under different scenarios.^[^ ^21^, ^30^ ^]^ To investigate the contribution of 1‐MNA to breast cancer progression, we stimulated NNMT‐absent SKBR3 cells with 1‐MNA and test its effect on cell‐cycle progression. When added into the culture medium, 1‐MNA rapidly enters the cytoplasm of SKBR3 cells and MCF7 cells (Figure S2A,B, Supporting Information). Under 1 mm 1‐MNA stimulation conditions, the concentration of 1‐MNA in SKBR3 and MCF7 cells closely resembled that in MDA‐MB‐231 cells and BT‐549 cells, inducing the cell‐cycle progression from G0/G1 phase to G2/M phase of SKBR3 cells (Figure S2A–C, Supporting Information; Figure 3D). Moreover, cell‐cycle arrest in G0/G1 phase caused by NNMT silencing in MDA‐MB‐231 cells could be partially rescued by 1‐MNA stimulation (Figure 3E). To assess the impact of 1‐MNA on breast cancer progression in vivo, we subcutaneously injected MCF‐7 cells into the rear flank of the nude mice and treated the tumor‐bearing mice with 1‐MNA and PBS, respectively. As depicted in Figure 3F, intraperitoneal injection of 1‐MNA resulted in a significant increase in tumor volume compared to the PBS‐treated group. This was consistent with our previous report that down‐regulation of NNMT expression in Bcap‐37 significantly inhibited tumor growth in vivo.^[^ ^26^ ^]^ All these collectively suggest that 1‐MNA is a biological active metabolite in promoting cell‐cycle progression of breast cancer.

Consistent with these findings, western‐blot data demonstrated that stimulation with 1‐MNA significantly down‐regulated p27 expression in both MCF7 and SKBR3 cells compared to untreated cells, while p53 and p21 expression remained unaffected (Figure 3G). These results collectively suggest that 1‐MNA might serve as the intermediate responsible for the cell‐cycle promoting effect of NNMT by downregulating p27 expression in breast cancer cells.

### 2.4 NNMT/1‐MNA Promote p27 Degradation via Increasing Cullin‐1 Neddylation

**Figure 4:**
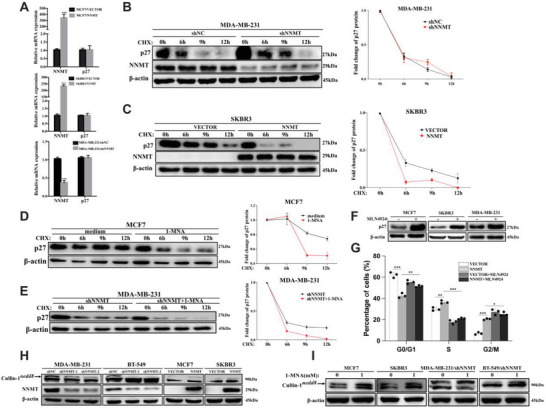
NNMT/1‐MNA promote p27 degradation via increasing cullin‐1 neddylation. A) qRT‐PCR analysis of p27 mRNA and NNMT mRNA transcription in indicated cells(MCF7/VECTOR and MCF7/NNMT, SKBR3/VECTOR and SKBR3/NNMT, MDA‐MB‐231/shNC and MDA‐MB‐231/shNNMT). B) Western‐blot analysis of protein level of p27 and NNMT in NNMT knockdown cells (MDA‐MB‐231/shNNMT) and C) NNMT over‐expression cell line (SKBR3/NNMT) at different time points (0 h, 6 h, 9 h, and 12 h) after CHX (30 µg mL^−1^) treatments, and its relative expression to β‐actin was calculated. D, MCF7, or MDA‐MB‐231 transfected with NNMT shRNA (MDA‐MB‐231/shNNMT)(E) were firstly stimulated with or without 1‐MNA(1.0 mm) for 24 h, then treated with CHX (30 µg mL^−1^) for indicated time point (0 h, 6 h, 9 h, and 12 h), then the protein level of p27 was detected using Western‐blot and its relative expression to β‐actin was calculated. F) western‐blot analysis of protein level of p27 in MCF7, SKBR3 and MDA‐MB‐231 cells treated with MLN4924 (600 nm for 24 h) or DMSO as indicated. G, flow cytometry analysis of cell‐cycle progression of SKBR3/VECTOR cells, NNMT‐over‐expressing SKBR3 cells (SKBR3/NNMT), and SKBR3/VECTOR and SKBR3/NNMT cells treated with MLN4924 (600 nm, 24 h), and the results were calculated as percentage of cells in G0/G1 phase, S phase and G2/M phase. H) Western‐blot detection of cullin‐1 and NNMT in NNMT over‐expression cell line (MCF7/NNMT, SKBR3/NNMT) and two distinct NNMT specific shRNA (shNNMT‐1 and shNNMT‐2) transfected cell line (BT‐549 and MDA‐MB‐231), empty vector or control shRNA transfected cell lines were used as control, and nedd8 conjugated cullin‐1 was indicated. I) indicated cells were stimulated with or without 1‐MNA(1.0 mm) for 24 h, protein level of cullin‐1 was detected using western‐blot and nedd8 conjugated cullin‐1 was indicated. Data were presented as mean ± SEM. *, *P*<0.05; **, *P*<0.01; ***, *P*<0.001.

To investigate the mechanism by which NNMT/1‐MNA regulate p27 expression, we performed qRT‐PCR analysis of p27 mRNA transcription in MCF7 and SKBR3 cells over‐expressing NNMT. As shown in **Figure** **4**A, over‐expressing NNMT didn't show any impact on p27 mRNA transcription, indicating that the negative regulatory role of NNMT on p27 expression isn't on transcriptional level. To verify if p27 protein degradation is mediated by NNMT in breast cancer, we treated breast cancer cells with cycloheximide (CHX) to block new protein synthesis. As shown in Figure 4B, silencing of NNMT in MDA‐MB‐231 cells apparently prolonged proteins half‐life of p27 comparing with that of NNMT‐competent cells. Vice versa, over‐expression of NNMT in SKBR3 significantly sped up p27 degradation comparing with vector transfected cells (Figure 4C). Consistently, stimulation of MCF‐7 cells and NNMT‐knockdown cells (MDA‐MB‐231/shNNMT) with 1‐MNA significantly reduced half‐life of p27 proteins comparing with the respective untreated cells (Figure 4D,E). All these indicated that in breast cancer NNMT/1‐MNA promote cell‐cycle progression mainly by increasing p27 degradation.

It has been well documented that p27 is one of the substrates of the cullin‐RING ubiquitin ligase, CRL1^SKP2^(a complex composed of cullin‐1, RBX1, SKP1, and SKP2), whose activity is tightly controlled by cullin‐1 neddylation.^[^ ^23^, ^31^ ^]^ This was further confirmed by our study that blocking of neddylation by MLN4924 treatment in MCF7, SKBR3, and MDA‐MB‐231 cells caused p27 protein accumulation and cell cycle arrest at G2/M phase (Figure 4F; Figure S3A,B, Supporting Information). And also, the cell‐cycle progression and cell proliferation induced by NNMT over‐expression in SKBR3 cells was greatly inhibited upon neddylation blocking by MLN4924 (Figure 4G; Figure S3C, Supporting Information). Regarding to the positive regulatory role of NNMT/1‐MNA on p27 degradation in breast cancer cells as demonstrated above, it's highly possible that there may be a potential link between NNMT/1‐MNA and cullin‐1 neddylation in breast cancer. To verify this, we manipulated NNMT expression level in breast cancer cell lines and tested neddylation status of cullin‐1 by western‐blot. Indeed, neddylated cullin‐1 significantly decreased when silencing NNMT expression in MDA‐MB‐231 and BT‐549 cells, and meanwhile over‐expression of NNMT in SKBR3 cells and MCF‐7 cells significantly increased cullin‐1 neddyation (Figure 4H). Importantly, other components of CRL1^SKP2^, such as RBX1, SKP1, and SKP2, remained unaffected (Figure S4A, Supporting Information). Furthermore, stimulation of NNMT‐absent cells (MCF‐7 cells and SKBR3) and NNMT‐knockdown cells (MDA‐MB‐231/shNNMT and BT‐549/shNNMT) with 1‐MNA significantly increased cullin‐1 neddylation (Figure 4I). This strongly suggested that, in breast cancer cells, NNMT/1‐MNA may accelerate p27 degradation by increasing cullin‐1 neddylation which is central to CRL1 E3 ligase activity.

### 2.5 NNMT/1‐MNA Promote Cullin‐1 Neddylation via Protecting UBC12 from Degradation

**Figure 5:**
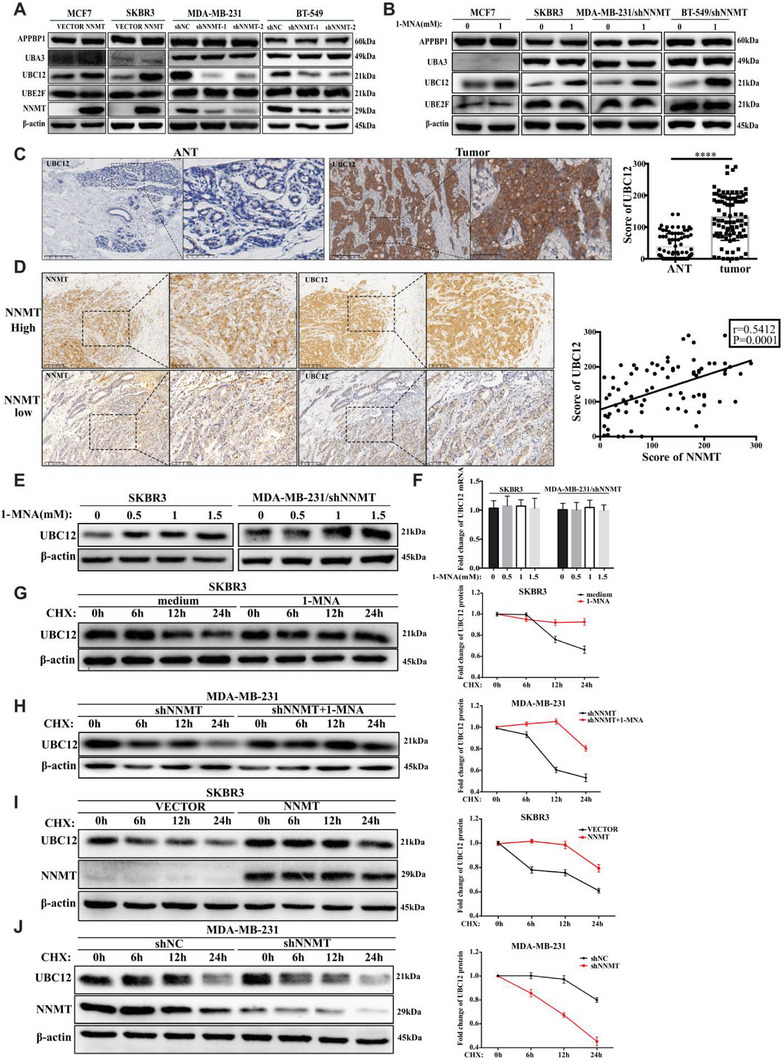
NNMT/1‐MNA promote Cullin‐1 neddylation via protecting UBC12 from degradation. A) Western‐blot detection of nedd8 activating E1 enzyme (APPBP1 and UBA3) and NEDD8‐conjugating E2 enzyme (UBC12 and UBE2F) in NNMT over‐expression cell line (MCF7/NNMT, SKBR3/NNMT) and two distinct NNMT specific shRNA (1# and 2#) transfected cell line (BT‐549 and MDA‐MB‐231), empty vector or non‐specific shRNA transfected cell lines were used as control. B) indicated cells (SKBR3, MCF7, MDA‐MB‐231/shNNMT and BT‐549/shNNMT) were stimulated with or without 1‐MNA (1.0 mm) for 24 h, then the protein level of APPBP1, UBA3, UBC12, and UBE2F was detected using western‐blot. C) IHC staining of human breast cancer tissue and adjacent normal tissue using specific antibodies against UBC12, and the expression level of UBC12 in the tissue was scored from 0 to 300 according to the staining intensity. D) IHC(Immunohistochemistry) staining of NNMT and UBC12 protein expression in human breast cancer tissue. Pearson correlation analysis of the association between NNMT and UBC12 in breast cancer tissue (*r* = 0.5412, *P* = 0.0001) was performed. E) SKBR3, MCF7, and MDA‐MB‐231/shNNMT were stimulated with increasing dose of 1‐MNA (0 mm, 0.5 mm, 1.0 mm, and 1.5 mm), and UBC12 protein level was detected by western‐blot in cell lysates. F) qRT‐PCR analysis of UBC12 mRNA transcription in SKBR3 and MDA‐MB‐231/shNNMT stimulated with increasing dose of 1‐MNA (0 mm, 0.5 mm, 1.0 mm, and 1.5 mm). G) SKBR3 or MDA‐MB‐231/shNNMT H) stimulated with or without 1‐MNA (1.0 mm, 24 h) were treated with CHX (30 µg mL^−1^) for indicated time points (0 h, 6 h,12 h, and 24 h), and then the protein level of UBC12 was detected using western‐blot and its relative expression to β‐actin was calculated. I) SKBR3 cells transfected with empty vector or NNMT‐over‐expressing plasmids J) MDA‐MB‐231 cells transfected with control shRNA and NNMT shRNA NNMT were treated with CHX (30 µg mL^−1^) for indicated time points (0 h, 6 h,12 h, and 24 h), and then the protein level of UBC12 was detected using western‐blot and its relative expression to β‐actin was calculated. Data are representative of three independent experiments. Data were presented as mean ± SEM. ****, *P*<0.0001.

Neddylation is an enzymatic cascade reaction involving E1 NEDD8‐activating enzyme(APPBP1‐UBA3), E2 NEDD8‐conjugating enzymes (UBC12 and UBE2F) and many E3s, which ultimately conjugate NEDD8 to specific substrates.^[^ ^23^, ^24^ ^]^ Then we wanted to determine whether the observed change of cullin‐1 neddylation is due to the impact of NNMT/1‐MNA on these neddylation‐associated E1 and E2 enzymes. As shown in **Figure** **5**a, introducing of NNMT into MCF7 and SKBR3 cells significantly increased protein level of UBC12, and interfering NNMT expression in MDA‐MB‐231 and BT‐549 cells specifically reduced UBC12 expression, without any effect on the protein level of E1(APPBP1‐UBA3) and the other E2(UBE2F). Meanwhile, 1‐MNA stimulation specifically increased protein expression of UBC12 in NNMT‐absent cells (MCF‐7 cells and SKBR3) and NNMT‐knockdown cells (MDA‐MB‐231/shNNMT and BT‐549/shNNMT), without any impact on the protein level of E1(APPBP1‐UBA3) and the other E2(UBE2F) (Figure 5B). Previous research demonstrated that UBC12 is E2 NEDD8‐conjugating enzyme required for cullin‐1‐4 neddylation, and UBE2F specifically participate in cullin‐5 neddylation.^[^ ^32^ ^]^ We further observed that, in addition to cullin‐1, interfering NNMT expression in MDA‐MB‐231 and BT‐549 cells noticeably reduced the neddylation of cullin‐2, cullin‐3, and cullin‐4a, while cullin‐5 neddylation remained unaffected (Figure S4B, Supporting Information). Conversely, overexpression of NNMT in NNMT‐absent cells (MCF‐7 cells and SKBR3) or 1‐MNA stimulation significantly increased the neddylation of cullin‐2, cullin‐3, and cullin‐4a, while cullin‐5 neddylation remained unaffected (Figure S4B,C, Supporting Information). This further corroborated the positive regulatory role of NNMT/1‐MNA on UBC12 expression and subsequent cullins neddylation. We then investigated UBC12 expression in breast cancer tissue using IHC study. The results showed that breast cancer tissues were significantly higher stained for UBC12 than that of adjacent normal tissue (Figure 5C). Notably, Pearson correlation analysis demonstrated a statistically significant positive correlation between the NNMT staining score and the UBC12 score (*r* = 0.5412, *P* = 0.0001) (Figure 5D). Therefore, it's likely that that in breast cancer NNMT/1‐MNA promote neddylation pathway activation via increasing UBC12 expression.

When 1‐MNA was added directly into the culture medium of NNMT‐absent cells (SKBR3 and MCF7) or NNMT‐knockdown cells (MDA‐MB‐231/shNNMT cells), the amount of UBC12 proteins but not UBC12 mRNA was increased upon 1‐MNA stimulation in a dose dependent manner (Figure 5E,F). These findings suggest the presence of unidentified mechanisms, beyond transcriptional regulation, that contribute to the UBC12‐promoting effect of NNMT/1‐MNA. Then, the stability of UBC12 protein was examined in breast cancer cells treated with CHX (Cycloheximide), an inhibitor of newly synthesized protein in eukaryotic cells. As shown in Figure 5G,H, the degradation process of UBC12 was significantly delayed when SKBR3 cells and NNMT‐knockdown cells(MDA‐MB‐231/shNNMT) were teated with 1‐MNA. Accordingly, the degradation process of UBC12 was delayed to 12 h after CHX treatment when NNMT was over‐expressed in SKBR3 cells (Figure 5I). Likewise, knock‐down of NNMT in MDA‐MB‐231(MDA‐MB‐231/shNNMT) greatly accelerated the degradation process of UBC12 in contrast to that of control shRNA treated MDA‐MB‐231(MDA‐MB‐321/shNC) (Figure 5J). Taken together, NNMT/1‐MNA could prolong protein half‐life of UBC12 in breast cancer cells, thereby increasing cullin‐1 neddylation

### 2.6 1‐MNA Inhibits the Localization of UBC12 to Lysosome

**Figure 6:**
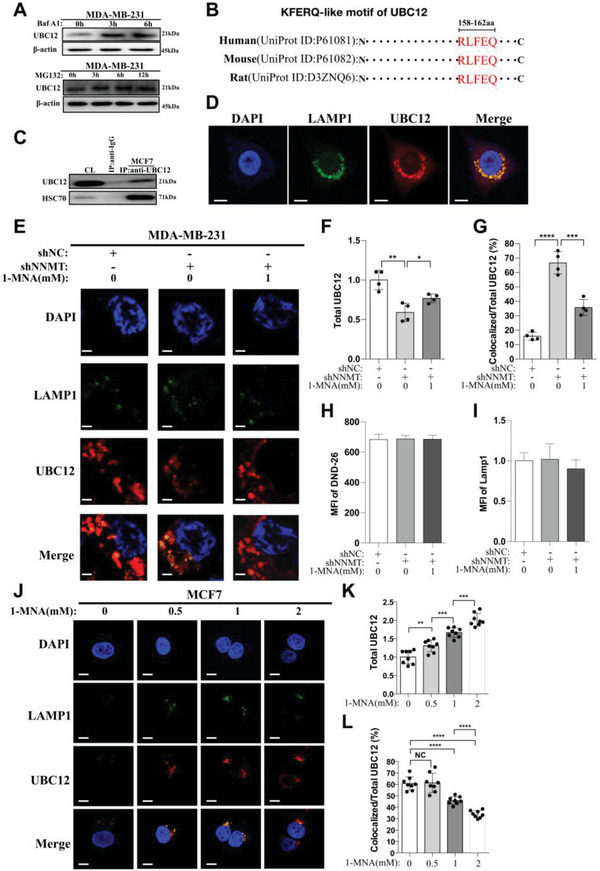
1‐MNA inhibit the localization of UBC12 to lysosome. A) western‐blot detection of protein level of UBC12 in MDA‐MB‐231 cells at different time points (0 h, 3 h, 6 h) after Bafilomycin A1(BafA1, 20 nm) treatments or at different time points (0 h, 3 h, 6 h, and 12 h) after MG132 (30ug mL^−1^) treatments. B) KFERQ‐like motif in UBC12 proteins analyzed using KFERQ finder V0.8. C) Anti‐UBC12 immunoblot analysis of HSC70 immunoprecipitates of lysates from MCF7 cells. D) Confocal microscopy imaging of UBC12 proteins and lysosome (LAMP1) in MDA‐MB‐231 cells, the bar represents 15 µm. E) Confocal microscopy imaging of UBC12 protein and lysosome (LAMP1) in MDA‐MB‐231/NC, NNMT‐silencing cells (MDA‐MB‐231/shNNMT) and MDA‐MB‐231/shNNMT cells treated with 1‐MNA (1 mm, 24 h), the bar represents 15 µm. Then total UBC12 F), percentage of UBC12 colocalized with LAMP1(lysosome) G) and abundance of lysosome (LAMP1) I) were calculated based on above confocal imaging. H) lysosome activity was analyzed in MDA‐MB‐231/NC, NNMT‐silencing cells (MDA‐MB‐231/shNNMT) and MDA‐MB‐231/shNNMT cells treated with 1‐MNA (1 mm, 24 h). J) Confocal microscopy imaging of UBC12 proteins and lysosome (LAMP1) in MCF7 cells treated with indicated concentration of 1‐MNA (0 mm, 0.5 mm, 1 mm, and 2 mm) for 24 h, the bar represents 15 µm. Then total UBC12 K) and percentage of UBC12 colocalized with LAMP1 among total UBC12 L) were calculated based on above confocal imaging. Data are representative of three independent experiments. Data were presented as mean ± SEM.*, *P*<0.05; **, *P*<0.01; ***, *P*<0.001; ****, *P*<0.0001.

The major protein degradation pathways in cells include the ubiquitin‐proteasome system (UPS) and autophagy‐lysosome pathway.^[^ ^33^ ^]^ To determine which pathway is mainly involved in UBC12 protein degradation in breast cancer, we respectively treated breast cancer cells with proteasome specific inhibitor (MG132) and lysosome specific inhibitor (Bafilomycin A1, BafA1) and monitored UBC12 protein level change. Surprisingly, only BafA1 treatment, not MG132, caused significant accumulation of UBC12 proteins (**Figure** **6**A). This indicated that lysosome was the destined degradation organelle of UBC12 proteins. In line with this, the KFERQ‐like motif, a sequence pattern present in proteins targeted for degradation through chaperone‐mediated autophagy (CMA),^[^ ^34^, ^35^ ^]^ is located between amino acid positions 158 and 162 in UBC12 proteins and is conserved among humans, mice, and rats (Figure 6B). Interaction of UBC12 with HSC70, a well‐known chaperone protein which binds KFERQ‐like motif and recruits the target proteins to lysosome,^[^ ^34^, ^35^ ^]^ was also confirmed in the immunoprecipitation assay (Figure 6C). More importantly, our confocal microscopy study performed in MDA‐MB‐231 cells showed that considerable proportion of cytosolic UBC12 proteins localized to lysosome (labeled by LAMP1) (Figure 6D). All these results provide the first line of evidence that in breast cancer cells UBC12 was mainly degraded through CMA‐lysosome pathway, other than the ubiquitin (Ub)–proteasome pathway.

Because UBC12 proteins were mainly targeted to lysosome for degradation as demonstrated above, we then visualized the intracellular localization of UBC12 upon 1‐MNA stimulation using confocal microscopy. Megascopically, silencing of NNMT in MDA‐MB‐231 cells significantly reduced the MFI of UBC12, which was then partially but significantly restored by 1‐MNA stimulation (Figure 6E,F). These were mirrored by the fact that 1‐MNA stimulation significantly inhibited the increase of the percentage of lysosome‐localized UBC12 caused by NNMT silencing in MDA‐MB‐231 cells (Figure 6E,G). And all the observed phenomena were not due to any change of lysosome itself, because neither silencing of NNMT nor 1‐MNA stimulation pose any impact on lysosome activity and lysosome abundance in MDA‐MB‐231 cells (Figure 6H,I). Furthermore, confocal microscopy analysis in MCF7 cells showed that 1‐MNA stimulation dose‐dependently increased the protein level of UBC12 and prevented the localization of UBC12 to lysosome (Figure 6J–L) comparing with that of untreated cells. Therefore, in breast cancer cell, 1‐MNA stabilize UBC12 protein by preventing its localization to lysosome for degradation.

### 2.7 UBC12 may be the Potential Binding Target of 1‐MNA

**Figure 7:**
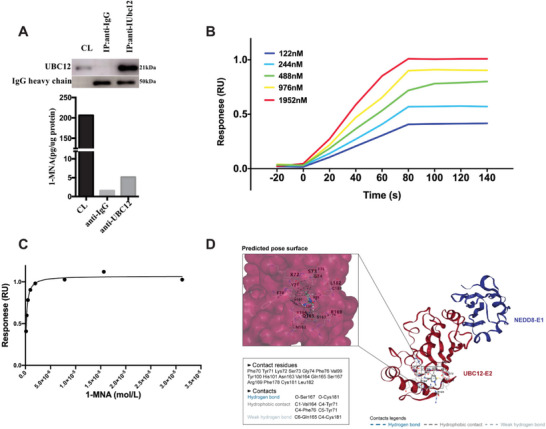
UBC12 may be the potential binding target of 1‐MNA. A) cell lysates of MDA‐MB‐231 cells was immunoprecipitated with anti‐UBC12 and IgG antibody respectively, then the immunoprecipitates were subjected to methanol extraction and SDS extraction, amount of 1‐MNA was determined by LC‐MS/MS and presence of UBC12 was determined by immunoblot. B,C) the binding affinity of 1‐MNA to UBC12 was evaluated via SPR as illustrated in material and methods, D) silico protein–ligand docking software was employed to analyze the binding affinities and modes of interaction between the1‐MNA and UBC12, the binding poses and interactions of 1‐MNA with UBC12 protein were obtained with CB‐Dock2 and the binding score for each interaction was generated. Data are representative of three independent experiments.

Protein to small molecule interaction (PMSI) has been recognized as one of the common ways of metabolites in regulating protein function.^[^ ^36^, ^37^ ^]^ This prompted us to explore if there is potential interaction between 1‐MNA and UBC12. First of all, we did cellular thermo shift assay (CETSA), in which presence of 1‐MNA lead to the decrease of thermo stability of UBC12 when thermo temperature was over 42 °C, suggesting that UBC12 may be the direct binding target of 1‐MNA (Figure S5, Supporting Information). To provide additional confirmation, we conducted an analysis to quantify the binding of 1‐MNA to UBC12. This analysis involved a combination of immunoprecipitation and mass spectrometry, a proven and effective method for identifying small metabolites associated with target proteins.^[^ ^38^ ^]^ In this experiment, UBC12 protein was immunoprecipitated from NNMT‐competent MDA‐MB‐231 cells using anti‐UBC12 antibody, and then metabolites bound to UBC12 were extracted and subjected to LC‐MS/MS analysis. As shown in **Figure** **7**A, 1‐MNA was significantly higher pooled in UBC12 immunoprecipitates comparing with that of IgG immunoprecipitates. In addition, we examined the binding of 1‐MNA to UBC12 protein by surface plasmon resonance (SPR) in which UBC12 protein was immobilized. The dose‐dependent binding of 1‐MNA to UBC12 was observed (Figure 7B). The binding interaction exhibited a fast association rate (within 80s) and a slow dissociation rate (Figure 7B), indicating that 1‐MNA presented strong affinity for UBC12. Furthermore, the response unites at equilibrium were plotted against the 1‐MNA concentration, and the dissociation constant (KD) was calculated by fitting these data to fit curves through non‐linear regression analysis. The results showed that 1‐MNA bound to UBC12 with a KD value of 168.3 nm (Figure 7C). By means of CB‐Dock2, an accurate protein‐ligand blind docking tool, we obtained the possible binding pose between 1‐MNA and UBC12 protein. Results showed that 1‐MNA may bind to the predicted cavity of UBC12 through two visible hydrogen bonds (O‐Ser167 and O‐Cys181) and four hydrophobic contacts (C1‐Val164, C4‐Tyr71, C4‐Phe76 and C5‐Tyr71) (Figure 7D). Taken together, it's highly possible that UBC12 may be the intracellular binding target of 1‐MNA in breast cancer cells.

## 3 Discussion

Dysregulation of the cell cycle is a hallmark of cancer, leading to uncontrolled cell division. The orchestration of the cell cycle is primarily controlled by cyclins, cyclin‐dependent kinases (CDKs) and cyclin‐dependent kinase inhibitors (CKIs).^[^ ^5^ ^]^ Genetic and epigenetic alterations of these cell‐cycle regulatory proteins lead to unchecked cell proliferation in numerous types of solid cancer, including breast cancer.^[^ ^39^ ^]^ Moreover, E3 ubiquitin ligases, such as Skp1–Cul1–F‐box‐protein (SCF) complex and the anaphase‐promoting complex/cyclosome (APC/C), play a significant role in the regulation of mitotic protein expression, thereby influencing cell cycle transitions.^[^ ^40^ ^]^ However, the regulatory effect of metabolic changes, which is also a common feature of breast cancer, on cell‐cycle progression hasn't been fully investigated.

Cancer cells have different metabolic features than normal cells, such as increased glucose consumption, altered TCA cycle activity, increased anabolic pathways, and altered redox balance.^[^ ^41^ ^]^ These metabolic changes are driven by both intrinsic factors, such as oncogenic mutations and signaling pathways, and extrinsic factors, such as nutrient availability and interactions with other cell types in the tumor microenvironment (TME).^[^ ^42^, ^43^ ^]^ The abnormal expression of NNMT in various human cancers has been widely reported and its positive correlation with several cancer features has gained significant attention over the past few decades. However, the precise biological role of NNMT in tumorigenesis, as an N‐methyl transferase, hasn't been fully understood. Our previous researches demonstrated that ectopic expression of NNMT in breast cancer not only promotes chemoresistance of breast cancer cells, but also contributes to high metastasis of TNBC by mediating membrane fluidity.^[^ ^17^, ^18^ ^]^ More recently, Joana et al. reported that NNMT sustains a core epigenetic program that promotes metastatic colonization in breast cancer.^[^ ^44^ ^]^ Our current study revealed that NNMT is a critical promoter of breast cancer cell proliferation and cell cycle progression, providing additional evidence for the role of NNMT in promoting breast cancer progression.

In mesenchymal cancer stem cells, overexpression of NNMT leads to depletion of intracellular NAM, resulting in increased PARP1 activity and enhanced resistance of cancer cells to chemoradiotherapy.^[^ ^16^, ^45^ ^]^ Ulanovskaya et al. reported that NNMT functions as a regulator of the methyl donor sink in cancer cells, and its overexpression leads to a 40–50% decrease in levels of H3K4me3, H3K9me2, and H3K27me3.^[^ ^46^ ^]^ Mark et al. demonstrated that high level of NNMT in CAFs resulted in the depletion of S‐adenosyl methionine and a decrease in histone methylation, leading to extensive gene expression changes in the tumor stroma.^[^ ^14^ ^]^ Although these studies on NNMT are performed in different types of tumors, the mechanisms suggest that NNMT regulates tumor‐associated protein modifications by depleting intracellular SAM and NAM, thereby promoting tumorigenesis. However, the biological role of its metabolite, 1‐MNA, has not been well explored. In the present study, by constructing NNMT enzyme activity dead mutants (NNMT‐Y20 and NNMT‐D197), we demonstrated that N‐methyl transferring activity of NNMT is required for its role in down‐regulating p27 expression and resultant cell‐cycle progression. Specifically, 1‐MNA was the responsible molecule that reduce p27 expression by decreasing its protein half‐life, other than its transcription. Recently, two publications reported that 1‐MNA exhibits inhibitory effect on IFN‐γ expression by T cells and the activation of NLRP3 inflammasome in human macrophages.^[^ ^21^, ^47^ ^]^ All these overturn the previous idea that 1‐MNA is a small inert metabolite excreted in urine. However, the underlying mechanism and intracellular target of 1‐MNA still remain elusive.

The research that 1‐MNA inhibit the ubiquitination and degradation of Sirt1 proteins indicates the potential regulatory role of 1‐MNA on post‐translational modifications(PTMs) of proteins.^[^ ^20^ ^]^ Here we found that 1‐MNA also paly a role on the stability of p27 proteins in breast cancer. After further exploration, it was discovered that 1‐MNA stimulation significantly increase cullin‐1 neddylation, activator of CRL1 E3 ubiquitin ligase, by increasing UBC12 proteins level. In line with this, we provided evidence that NNMT/1‐MNA enhances the overall neddylation levels of the other three cullins proteins(cullin‐2,3,4), which are also well‐known substrates of UBC12.^[^ ^32^ ^]^ These findings confirm NNMT/1‐MNA as a positive upstream regulator of the neddylation pathway in breast cancer. Besides cullins (cullin1‐cullin5) family, more and more proteins such as P53, BCA3, MDM2, VHL, and PCNA have been demonstrated to be subjected to neddylation and their neddylation showed biological relevance to tumorigenesis.^[^ ^48^, ^49^, ^50^ ^]^ NEDD8 expression, which represents general neddylation status, was reported to be more prominent in ER‐positive breast cancer and be involved in regulating ERα expression.^[^ ^51^ ^]^ Considering the regulatory role of NNMT/1‐MNA on UBC12 protein levels, it is plausible that NNMT/1‐MNA is involved in the regulation of protein stability within the cells. Therefore, we believe that the role of NNMT/1‐MNA in breast cancer is not limited to cell cycle regulation and tumor metastasis.

UBC12, one of the two NEDD8‐conjugating enzyme E2s identified as far, has been assumed as an alternative therapeutic target for neddylation pathway inactivation in lung cancer.^[^ ^52^ ^]^ Transcriptional regulation of UBC12 mRNA expression by HIF‐1α and c‐jun has been reported in lung cancer cells in response to stress.^[^ ^32^ ^]^ However, how the degradation process or protein stability of UBC12 is regulated in cancer cells has never been disclosed. Before resolving these issues, we evaluated the protein half‐life of UBC12 in breast cancer cells and identified UBC12 as a long‐lived intracellular protein with a half‐life around 24 hours. Surprisingly, UBC12 protein bind to HSC70 and localize to lysosome as its destined degradation organelle, which is consistent with the concept that some long‐lived proteins tend to be degraded in lysosome.^[^ ^53^, ^54^ ^]^ This is the first line of evidence that UBC12 is a long‐lived protein that is targeted to lysosome for degradation. More importantly, we demonstrated that the increased expression of UBC12 induced by NNMT/1‐MNA was due to its role in stabilizing UBC12 proteins, other than increasing UBC12 mRNA transcription.

A high concentration of 1‐MNA was detected in the supernatant of NNMT‐competent breast cancer cells (Figure S2D–F, Supporting Information). 1‐MNA present in the culture medium was easily taken into the cytosol by NNMT‐absent cells, including breast cancer cells (MCF7 and SKBR3), and the amount of 1‐MNA intake by cells increased with the increment of extracellular level of 1‐MNA stimulants (Figure S2A,B, Supporting Information). These suggest that transport of 1‐MNA is mainly passively driven by the concentration gradient pressure of 1‐MNA inside and outside the cells. This indicate that once 1‐MNA is present, it may exert biological effect on NNMT‐absent cancer cells. Therefore, even the restricted expression of NNMT to specific cells, the biological effects of this enzyme can be significantly amplified by the release of its bioactive metabolite 1‐MNA. More convincingly, the UBC12‐stabilizing effect of 1‐MNA was also applicable to NNMT‐absent colon cancer cells and 293T cells (Figure S6A,B, Supporting Information). In some of the breast cancer tissues included in this study, we noticed that the stroma cells were also stained positive for NNMT (Figure S7, Supporting Information), which is consistent with previous studies in colon cancer and ovarian cancer.^[^ ^21^, ^30^ ^]^ From this aspect, both NNMT‐positive stroma cells and tumor cells are considered as main cellular source of 1‐MNA present in TME, which then serve as a signaling molecule transducing UBC12‐stabilizing signal from NNMT‐competent cells to adjacent NNMT‐free cells. Besides, 1‐MNA released from NNMT‐expressing breast cancer tissues may exert minimal influence on the circulating level of 1‐MNA, since we didn't observe significant difference about serum level of 1‐MNA between breast cancer patients and healthy control (Figure S8, Supporting Information). In other words, the concentration of 1‐MNA in the serum may not accurately reflect the 1‐MNA levels in breast cancer tissue.

Although antithrombotic, anti‐inflammatory and gastroprotective roles of 1‐MNA have been reported by previous studies,^[^ ^55^, ^56^, ^57^ ^]^ the underlying functional mechanism or the intracellular responder elements of 1‐MNA have not been clarified yet. When looking at the mechanism by which 1‐MNA prevents UBC12 degradation in lysosome, we surprisingly found that UBC12 may be the potential binding target of 1‐MNA, which was evident both in SPR assay and IP‐LC‐MS/MS assay. Based on this alone, we cannot make the conclusion that binding of 1‐MNA to UBC12 is indispensable for its role in UBC12 stabilization. But this study at least gives us an important clue that we can follow when investigating the functional mechanism of 1‐MNA. Therefore, clarifying biochemical properties of the binding between 1‐MNA and UBC12, and identifying other potential intracellular protein targets of 1‐MNA, will fully illustrate the biological roles of 1‐MNA and provide novel therapeutic options in cancer treatment.

According to these results, we clarified that NNMT/1‐MNA is a positive upstream regulator of the neddylation pathway in breast cancer. Any change of the enzymatic cascade of NEDD8‐activating enzyme E1 and NEDD8‐conjugating enzyme E2s would cause profound influence on the neddylation status of downstream substrate proteins.^[^ ^23^, ^32^ ^]^ This biological role of NNMT on neddylation pathway activation was partly reflected on the fact that in breast cancer cells NNMT promotes cell‐cycle progression by reinforcing neddylation‐mediated degradation of p27. Given the extensive effect of the neddylation pathway on various cellular biological behaviors, whether chemoresistance‐promoting, migration‐promoting and apoptosis‐inhibiting roles of NNMT demonstrated in breast cancer^[^ ^17^, ^18^, ^26^ ^]^ are partly dependent on its effect on the neddylation pathway is worth reinvestigating. However, we believe that other unknown factors may also participate in neddylation pathway activation in breast cancer, because NEDD8‐activating enzyme E1(NAE1), whose expression wasn't regulated by NNMT, was also reported to be highly expressed in breast cancer tissue compared with ANT.^[^ ^51^ ^]^

Collectively, we verified that high expression of NNMT in breast cancer cells contributes to cell‐cycle progression, cell proliferation, and serves as a poor prognostic factor in breast cancer. In terms of mechanism, 1‐MNA, NNMT metabolic product, protect UBC12 from degradation, thereby activating neddylation‐mediated degradation of p27 proteins. These findings suggest that breast cancer cells expressing NNMT may potentially enhance the proliferation of neighboring NNMT‐negative cancer cells by releasing 1‐MNA into the tumor microenvironment (TME). Given the findings of this study and the previously reported metastasis‐promoting role of NNMT in breast cancer, it is crucial and significant to develop diagnostic kits for the detection of NNMT in breast cancer and to explore NNMT inhibitors for therapeutic purposes in breast cancer.

## 4 Experimental Section

### Reagents, Antibodies, and Cell Lines

MLN4924(HY‐70062) and Bafilomycin A1(BafA1)(HY‐100558) were purchased from Med Chem Express. Cycloheximide (CHX)(S7418) and MG132(S2619) was purchased from Selleck (Selleck, Shanghai, China).1‐Methylnicotinamide chloride(1‐MNA) was purchased from Sigma (#1005‐24‐9, Sigma‐Aldrich). The mouse anti‐human NNMT monoclonal antibody(clone:1E7) was prepared through the hybridoma technique as previously described.^[^ ^26^ ^]^ The anti‐β‐actin (#4970), anti‐p53(#2524), anti‐p21(#2947), anti‐p57(#2557), anti‐CDK2(#2456), anti‐CDK4(#12 790), anti‐Cyclin E1(#4129), anti‐Cyclin D1(#2978), anti‐E2F‐1(#3742), and anti‐p27 (#3686) were obtained from Cell Signaling Technology (CST, Beverly, Massachusetts, USA). The anti‐cullin1∼5 (ab75817; ab166917; ab75851; ab92554; ab184177), anti‐APPBP1(ab187142), anti‐UBA3(ab247153), anti‐UBC12 (ab109507), anti‐HSC‐70(ab51052), anti‐Rb(ab181616) and anti‐UBE2F (ab185234) were obtained from Abcam (Abcam, Cambridge Science Park, Cambridge, England). Anti‐Ki‐67(clone:GM027) was obtained from Gene Tech Company (Shanghai).

Human breast cancer cell lines, including MCF7, SKBR3, MDA‐MB‐231, BT‐549, T47D, MDA‐MB‐468, Bcap37 and HCC1937, were purchased from American Type Culture Collection (ATCC, USA). Human MCF7, SKBR3, MDA‐MB‐231, MDA‐MB‐468, T47D and Bcap37 were cultured in DMEM (Gibco, Grand Island, NY, USA). HCC1937 cells were grown in RPMI 1640 medium (Gibco). BT‐549 cells were cultured in RPMI 1640 medium containing 0.023 U/mL insulin. All mediums were supplemented with 10% fetal bovine serum (Gibco, Long Island, NY, USA), 100U mL^−1^ penicillin (Sigma‐Aldrich, St. Louis, MO, USA) and 100 mg mL^−1^ streptomycin (Sigma‐Aldrich, St. Louis, MO, USA). The cells were cultured at 37 °C with 5% CO2.

### Patient's Information

Human breast cancer tissues were from pathology department, Sir Run Run Shaw hospital, Hangzhou, China. Tissue arrays were from 90 patients with histologically confirmed primary breast cancer who underwent mastectomy between 2011 and 2018. Their clinicopathologic characteristics information was available. This study was approved by the Human Research Ethics Committee of Sir Run Run Shaw Hospital (Permit Number: 20180601–006). All patients provided written informed consent before their inclusion in this study.

### IHC Staining and Evaluation

Sections of formalin‐fixed, paraffin‐embedded tumor specimens were deparaffinized in xylene and hydrated in a graded alcohol series. Endogenous peroxidase was blocked using 1% H_2_O_2_ for 5 min. For NNMT, Ki‐67, UBC12 and p27 staining, antigens were retrieved with citrate buffer (10 mm, pH 6.0) for 15 min at 100 °C in a microwave oven. Then, the sections were washed with ddH_2_O and blocked with 1% bovine serum albumin (BSA) for 30 min, and then incubated with primary antibodies, separately (NNMT,1:1400; UBC12,1:250, p27,1:400, Ki‐67, 1:600) at room temperature in a moist chamber for 40 min. After being washed with PBS for three times, sections were incubated with secondary antibodies (HRP rabbit/mouse En Vision) for 30 min. After secondary antibody staining, the sections were washed with PBS for three times and incubated with diaminobenzidine (DAB) for 5 min. Then, the nuclei were stained with hematoxylin. Staining results were evaluated independently by two pathologists without prior knowledge of clinicopathologic data.

The expression of NNMT, UBC12, and p27 was scored using the semi‐quantitative H‐score method, which was based on the intensity of staining and the percentage of stained cells. Briefly, the staining intensity of each sample was graded as 0 (no staining), 1+ (weak staining), 2+ (moderate staining), or 3+ (intense staining). Then the percentage of stained cells (0‐100%) was multiplied by the intensity grade (0–3) to yield an intensity percentage score. The staining scores were then calculated from the sum of the four intensity percentage scores. Therefore, the overall semi‐quantitative score ranged from 0 (no staining) to 300 (100% of cells with 3+ staining intensity).

### Public Cohorts’ Collection and Data Analysis

A total of ten cohorts with bulk RNA transcripts were used to identify the prognostic values of NNMT in breast cancer patients in the public GEO repository. Among them, eight cohorts were collected with overall survival data (GSE103091, GSE16446, GSE20685, GSE20711, GSE24450, GSE48391, GSE58812, GSE9893), and six cohorts were collected with Recurrence‐free survival (RFS) data (GSE19615, GSE20685, GSE20711, GSE9195 and GSE9893). The cut‐off of NNMT in each cohort was used to the “survival” package in R to find the best Youden index, then the log‐rank test and the cox‐regression analysis were performed according to the data types. A single‐cell RNA transcripts cohort (GSE176078, Chromium, 10X Genomics) from 26 Breast cancer samples with different TNBC status was also collected to check the expression of NNMT in different cell types). MAESTRO R package was used to annotate the major cell types of information of single cells. Tumor cells were grouped into NNMT high group and NNMT low group according to the expression of NNMT in scRNA data. By using Cellchat R packages, the cell‐to‐cell communications between tumor cells and immune cells were identified. The prognostic value of NNMT protein expression in breast cancer patients was analyzed using dataset (Liu‐2014, *n* = 126) collected in Kaplan‐Meier Plotter(http://kmplot.com/analysis).

### Over‐Expression and Knockdown of NNMT

The establishment of NNMT over‐expression cell lines were described in our previous paper.^[^ ^26^ ^]^ Briefly, human NNMT cDNA(NCBI Reference Sequence: NM_0 013 72045.1)was cloned and constructed into pcDNA3.1 plasmids. MCF7 and SK‐BR‐3 cells were transfected with pcDNA3.1/NNMT or empty pcDNA3.1 using Lipofectamine 3000, respectively. Then, the transfected cells were grown in complete medium containing 800 mg L^−1^ geneticin (G418; Gibco, Grand Island, NY, USA). After 2 weeks selection, positive colonies were picked and evaluated for NNMT expression by real‐time quantitative PCR and western‐blot. MCF7/Vector, SKBR3/Vector controls and MCF7/NNMT, SKBR3/NNMT with stable NNMT over‐expression were selected for further analysis.

Lentiviral vectors against NNMT were synthesized by GeneChem Co. Ltd (Shanghai, China). Table S1 (Supporting Information) showed the sequences of NNMT shRNA 1#, NNMT shRNA 2# and shRNA NC. For NNMT knockdown, MDA‐MB‐231 and BT‐549 cells were seeded (3×10^5^ cells per well) in six‐well plates and incubated for 24 h. When the cells reached 30–50% confluence, lentivirus containing shRNAs (NNMT shRNA 1#, NNMT shRNA 2# or shRNA NC) were added, respectively. After co‐culturing with lentivirus for 12 h, the supernatant was exchanged with fresh medium. The transduced cells were sorted using BD FACS Aria II system (BD Biosciences, San Jose, CA, USA) to obtain the GFP‐positive cell populations at 48 h after transfection and these populations were collected for subsequent analysis. Cells infected with shRNA NC were used as the negative control.

### Construction of NNMT Mutation Cell Line

As previous reported, substitution of either of the two site residues of human NNMT (D197 and Y‐20) would lead to more than 90% loss of its N‐methyl transferring activity.^[^ ^29^ ^]^ The site‐specific mutation sequence for D197 and Y‐20 were synthesized (Vigenebio, China) and listed in Figure S1 (Supporting Information). To generate lentivirus for NNMT over‐expression, we construct wide type NNMT (NCBI Reference Sequence: NM_0 013 72045.1), D197 and Y20 site mutation of NNMT into pLenti‐RFP‐Blasticidin‐CMV plasmid (Vigenebio, China), respectively. Then the pLenti‐RFP‐Blasticidin‐CMV plasmids loaded with WT‐NNMT, NNMT‐D197 and NNMT‐Y20 were transfected into 293T cell with PMG2D and psPAX2. 3 days later, the supernatant of 293T cell containing lentivirus was collected and used to transfect MCF7 cell. RFP positive cells sorted using BD FACS Aria II system (BD Biosciences, San Jose, CA, USA) and tested for NNMT expression.

### Cell Proliferation Assay

The cells proliferation was assessed by Cell‐Counting Kit‐8 (CCK8) colorimetric assay (CK04, Dojindo Laboratories, Japan). Briefly, cells were seeded in a 96 well flat‐bottomed microtiter plate at a density of 5×10^4^ cells mL^−1^ in 100 µL medium. Then, 10 µL CCK8 was added into each well and the cells were incubated at 37 °C for additional 2 h. The absorbance was measured at 450 nm using a microplate reader (Multiskan GO, Thermo Scientific). The experiment was repeated at least three times.

### MCF7 Xenograft Mouse Model

The animal experiment was approved by the Institutional Animal Care and Use Committee of Sir Run Run Shaw Hospital(Permit Number: 20210210–222). All procedures were performed in accordance with the ethical guidelines of the Declaration of Helsink. Six‐week‐old male BALB/c nude mice were purchased from Model Animal Research Center of Nanjing University and housed under pathogen‐free conditions with free access to food and water. All mice were acclimated for at least 5–7 days before treatment. Then each mouse was injected subcutaneously with 4×10^6^ MCF‐7 cells which were suspended in 100ul PBS. After approximately 10 days post the initial injection, mice were randomly assigned into 2 groups and treated with 1‐MNA (500 mg kg^−1^) or vehicle intraperitoneally every 2 days for 2 weeks. At the end of the experiment, all the mice are euthanized and all tumors were harvested. Tumor volume was calculated according to V = (length × width^2^)/2.

### Cell Lysate Thermal Shift Experiment

The cell lysate thermal shift experiment was performed as previous reported.^[^ ^58^ ^]^ Briefly, MCF7 cells were harvested and washed with Hank's balanced salt solution (HBSS), then resuspended in lysis buffer with complete protease inhibitor cocktail. The cell suspensions were freeze‐thawed three times using liquid nitrogen and passed through a 27″ gauge needle five times. The lysate was centrifuged at 20000 × g for 20 min at 4 °C, then was diluted with 200 µM dUMP lysis buffer and divvied into two aliquots. One aliquot was treated with 1‐MNA and the other aliquot with H_2_O (control). After 30 min incubation at room temperature, the lysates were divided into smaller (20 µm) aliquots and heated individually at four different temperatures for 5 min in a thermostatic metal bath (Hangzhou Bori Inc.). The heated lysates were centrifuged at 20000 × g for 20 min at 4 °C in order to separate the soluble fractions. The supernatants containing the soluble proteins were transferred to new 0.6 mL microtubes and analyzed by sodium dodecyl sulfate polyacrylamide gel electrophoresis (SDS‐PAGE) followed by western blot analysis.

### siRNA Mediated Knockdown Experiments

p27 siRNA (siG10514125225‐1‐5) and nonsense siRNA was obtained from RiboBio (Guangzhou RiboBio Co., China). MDA‐MB‐231 cells (3×10^5^ cells per well) were plated in 6 wells and were transiently transfected with 2 µg of siRNAs plus 4 µL Lipofectamine 2000 according to the manufacturer's instruction. 72 h later, cells were harvested.

### Cell Cycle Analysis by Flow Cytometry

The cell cycle analysis was carried out by flow cytometry using PI staining. After treated with DMSO or MLN4924 for 24 h, cells were harvested by 1000 rpm centrifugation for 5 min and fixed in ice‐cold 70% methanol overnight at −20 °C. The fixed cells were washed twice with cold PBS, and incubated with a PI working solution (CCS012, Multi Sciences Biotechnology, Hangzhou, China) for 30 min at room temperature. Cell cycle distribution was analyzed by flow cytometry (FACS Calibur flow cytometer, BD). ModFit LT software was used to analyze the results. Each experiment was performed at least three times.

### Real‐Time Quantitative PCR

Real‐time quantitative PCR analysis was conducted using the NovoStart SYBR qPCR SuperMix Plus Kit (Novoprotein, Shanghai, China). According to the protocol, total RNA was isolated using TRIzol reagent (Invitrogen, Carlsbad, CA, USA) and reverse transcribed into cDNA using HiFiScript cDNA Synthesis Kit (CWBio, Beijing, China). All primers were ordered from Invitrogen and the sequences of the primers are shown in Table S2 (Supporting Information). qPCR amplifications were performed using a Light Cycler 480 Real‐Time PCR system (Roche, USA). All experiments were independent and conducted at least three times. The mRNA levels of targeted genes were calculated using β‐actin as normalization control. The data was analyzed using the comparative threshold cycle (2^−∆∆Ct^) method.

### Western‐Blot Analysis

Cells were harvested, washed twice by PBS, lysed with RIPA (P0013B, Beyotime, Shanghai, China), then centrifuged 13 000 rpm for 20 min and the supernatants were collected. Protein concentration was detected by BCA assay kit (PQ0012, Multi Sciences Biotechnology, Hangzhou, China) according to the manufacturer's instructions. Equal amounts of protein (40 µg) were separated by SDS‐PAGE and transferred into PVDF membrane (ISEQ00010, Millipore, USA). Then, the membranes were blocked with 5% milk at room temperature and incubated with corresponding primary antibodies at 4 °C overnight, followed by incubation with corresponding HRP‐linked secondary antibodies (CST, Beverly, Massachusetts, USA) for 1 h at room temperature. Housekeeping product β‐actin served as a control for equal loading. Finally, the membranes were soaked with high sensitivity chemiluminescence detection reagents (FD Bioscience, Hangzhou, China), and imaged using Image Lab (BIO‐RAD, Hercules, CA, USA). The band intensity was quantitated using the software Image Lab. For protein half‐life analysis, the cells were treated with Cycloheximide(CHX) by time course before being subjected to RIPA buffer.

### Immunoprecipitation

For CO‐IP studies, MCF7 cells were prepared with IP lysis buffer (Beyotime, Shanghai, China), 800 µg proteins were incubated with 8 µg antibody against HSC70 overnight at 4 °C followed by incubation with 40 µl protein A+G agarose (Beyotime, Shanghai, China) for 4 h at 4 °C and the pellets were washed thoroughly with cold PBS. Immunoprecipitates were eluted from the beads by boiling in 40 µL 2× SDS‐PAGE loading buffer for 5 minutes and subjected to Western blot analysis.

### Confocal Microscopy

Cells were imaged using a confocal microscope (Olympus FluoView 1000, Tokyo, Japan). MDA‐MB‐231 cells were seeded in 4‐well chamber slider at a density of 2×10^4^ cells per well and incubated overnight. Cells were fixed with 4% paraformaldehyde in PBS, treated with 0.2%Triton X‐100 in PBS on ice for 10 min and then blocked with 1% BSA in PBS. After blocked, cells were washed twice with PBS and stained with anti‐UBC12 (ab109507, 1:200) and anti‐LAMP1 with FITC (cst58996S, 1:50) in the dark for 2 h at room temperature followed by three washes with PBS. Alexa Fluor 647 conjugated goat anti‐rabbit IgG H&L (ab150079, 1:500) was used as a secondary antibody. The cell culture was imaged immediately using confocal microscope (λEX = 488 and 559 nm for FITC tag and Alexa Fluor 594 excitation, respectively) using the 60× oil immersion objective. Colocalization analysis was performed using Image J (NIH, open‐source software, Bethesda, MD, USA).

### Quantification of 1‐MNA using LC‐MS/MS

The intracellular and extracellular 1‐MNA were analyzed by LC‐MS/MS method. AB SCIEX Triple Quad 4500MD mass spectrometry system was employed for this analysis. 100uL of biological cell samples or standards(1‐MNA) was added with 1% zinc sulfate solution to precipitate protein, then shaken with shaker at 400 rpm for 30 min, centrifuged at 14 000 rpm for 15 min, the supernatant was used for mass spectrometry analysis. Samples (5 µL) were injected onto Eclipse XDB‐C18 (4.6×150 mm, 5 µm; Agilent, USA) column connected to Jasper (SCIEX, USA) LC system. The isocratic mobile phase, a mixture of 0.1% formic acid and methanol mixture (v/v) was filtered through a 0.22 µm membrane filter (Millipore, USA) and then degassed ultrasonically for 15 min was delivered at a flow rate of 1 mL min^−1^ into the mass spectrometer electrospray ionization (ESI) chamber. Quantitation was achieved by MS/MS detection in positive ion mode for 1‐MNA and internal standard (N‐MNA‐d4). Detection of the ions was performed in the multiple reaction monitoring (MRM) mode, monitoring the transition of the m/z 137 precursor ion to the m/z 94 product ion for 1‐MNA and m/z 141 precursor ion to the m/z 84 product ion for N‐MNA‐d4 (internal standard). The retention times of 1‐MNA and N‐MNA‐d4 were 1.31 minutes and 1.65 minutes, respectively.

### LysoTracker Green DND‐26 Labeling and FACS Analysis

LysoTracker Green dye stains cellular acidic compartments and visualizes enlarged lysosomes. MDA‐MB‐231 cells were stained with LysoTracker Green (Cell Signaling Technology, MA, USA) according to the manufacturer's instructions by incubating them with the dye for 30 min at 37 °C. The fluorescence intensity was measured by flow cytometry (λEx = 488 nm and λEm = 507 nm).

### Molecule Docking Analysis

To analyze the binding affinities and modes of interaction between the drug candidate and their targets, CB‐Dock2, a silico protein–ligand docking software was employed.^[^ ^59^ ^]^ The molecular structure of 1‐MNA was retrieved from PubChem Compound (https://pubchem.ncbi.nlm.nih.gov/). The 3D coordinates of UBC12 (PDB ID, 1Y8X; resolution, 2.5 Å) was downloaded from the PDB online database. For docking analysis, all protein and molecular files were uploaded to CB‐Dock2 with all water molecules excluded and polar hydrogen atoms were added. Cavity prediction was first evaluated and then blind docking was performed for predicted cavity on UBC12‐E2 chain.

### Surface Plasmon Resonance (SPR)

Biacore T200 instruments (Cytiva) were used to evaluate the binding affinity of 1‐MNA to Ubc12 via SPR, as reported in the paper.^[^ ^60^ ^]^ Briefly, UBC12 were immobilized on the surface of CM5 chip by using amine‐coupling approach at a flow rate of 10 µL min^−1^ in 10 mm sodium acetate buffer (pH 4.0). The sensor surface was activated with a 7 min injection of the mixture of 50 mm N‐hydroxysuccinimide (NHS) and 200 mm 1‐ethyl‐3‐(3‐dimethylaminopropyl)carbodiimide (EDC). Then 20 µg mL^−1^ of UBC12 was injected to reach the target level of 3028 RU and the surface was blocked with 1 m ethanolamine, pH 8.5. Series concentrations of 1‐MNA were injected into the flow system and analyzed for 90 s, and the dissociation was 90 s. All binding analysis was performed in phosphate buffered saline (PBS) with 0.05% (v/v) Tween‐20, pH 7.4, at 25 °C. Prior to analysis, double reference subtractions and solvent corrections were made to eliminate bulk refractive index changes, injection noise, and data drift. The binding affinity was determined by fitting to a Langmuir 1:1 binding model within the Biacore Evaluation software (Cytiva).

### Statistical Analysis

Data of IHC analysis were expressed as mean ± standard deviation (SD). Data in vitro were expressed as mean ± standard deviation (SEM). All the analyses were performed with Graphpad Prism 6. The Student's test was used to determine the statistical significance of differences between comparison groups in vitro. The relationships between NNMT, UBC12 or p27 expression and clinicopathological attributes were analyzed using the Pearson's χ2 test. Survival rates were calculated using the Kaplan‐Meier method and compared using the log‐rank test. p values of less than 0.05 were considered statistically significant.

## Conflict of Interest

The authors declare no conflict of interest.

## Author Contributions

Y.M., X.H., and Y.W. contributed equally to this work.YL.M. and Ju.Z. performed conception and designing.YL.M., XC.H., JW.Y., J.Y., YD.C., YZ.W. performed methodology.XC.H., J.Y., YJ.L., YL.M., YZ.W., HT.Y., M.Y., YD.C., F.Z., GL.L., and J.Z. performed acquisition of data (cell line experiments, IHC staining, provide facilities, etc.).YL.M., J.Z., YZ.W., XC.H., SB.Y., and YZ.G. performed analysis and interpretation of data (e.g., statistical analysis, biostatistics, computational analysis).YL.M., J.Z., YZ.W., XC.H., and XY.X. performed writing, reviewing, and revision of the manuscript.J.Z., J.Y., and XY.X. helped in administrative, technical, or material support(i.e., reporting or organizing data, constructing databases).J.Z. and XY.X. performed study supervision.

## Supporting information

Supporting Information

Supplemental Table 3

Supplemental Table 4

## Data Availability

The data that support the findings of this study are available in the supplementary material of this article.
