# Supplementary material for: NNMT/1‐MNA Promote Cell‐Cycle Progression of Breast Cancer by Targeting UBC12/Cullin‐1‐Mediated Degradation of P27 Proteins
Source: Adv Sci (Weinh). 2023 Dec 21;11(9):2305907. doi: 10.1002/advs.202305907 (PMC10916551; doi:10.1002/advs.202305907)
Supplement: Supplementary file 1 — Supporting Information [file ADVS-11-2305907-s002.pdf]

ADVANCED SCIENCE
Open Access

Supporting Information

for *Adv. Sci.*, DOI 10.1002/advs.202305907

NNMT/1-MNA Promote Cell-Cycle Progression of Breast Cancer by Targeting UBC12/Cullin-1-Mediated Degradation of P27 Proteins

Yilei Ma, Xucheng Huang, Yanzhong Wang, Yinjiao Lei, Jinwei Yu, Shaobo Yu, Yuzhen Gao, Jun Yang, Feng Zhao, Haitao Yu, Jin Zeng, Yadong Chu, Min Yang, Guoli Li, Xinyou Xie and Jun Zhang**

```
CLUSTAL O(1.2.4) multiple sequence alignment


NNMT-Y20        atggaatcaggcttcacctccaaggacacctatctaagccattttaaccctcgggatgcc      60
NNMT-D197       atggaatcaggcttcacctccaaggacacctatctaagccattttaaccctcgggattac      60
WT-NNMT         atggaatcaggcttcacctccaaggacacctatctaagccattttaaccctcgggattac      60
                *********************************************************  *

NNMT-Y20        ctagaaaaatattacaagtttggttctaggcactctgcagaaagccagattcttaagcac      120
NNMT-D197       ctagaaaaatattacaagtttggttctaggcactctgcagaaagccagattcttaagcac      120
WT-NNMT         ctagaaaaatattacaagtttggttctaggcactctgcagaaagccagattcttaagcac      120
                ************************************************************

NNMT-Y20        cttctgaaaaatcttttcaagatattctgcctagacggtgtgaagggagacctgctgatt      180
NNMT-D197       cttctgaaaaatcttttcaagatattctgcctagacggtgtgaagggagacctgctgatt      180
WT-NNMT         cttctgaaaaatcttttcaagatattctgcctagacggtgtgaagggagacctgctgatt      180
                ************************************************************

NNMT-Y20        gacatcggctctggccccactatctatcagctcctctctgcttgtgaatcctttaaggag      240
NNMT-D197       gacatcggctctggccccactatctatcagctcctctctgcttgtgaatcctttaaggag      240
WT-NNMT         gacatcggctctggccccactatctatcagctcctctctgcttgtgaatcctttaaggag      240
                ************************************************************

NNMT-Y20        atcgtcgtcactgactactcagaccagaacctgcaggagctggagaagtggctgaagaaa      300
NNMT-D197       atcgtcgtcactgactactcagaccagaacctgcaggagctggagaagtggctgaagaaa      300
WT-NNMT         atcgtcgtcactgactactcagaccagaacctgcaggagctggagaagtggctgaagaaa      300
                ************************************************************

NNMT-Y20        gagccagaggcctttgactggtccccagtggtgacctatgtgtgtgatcttgaagggaac      360
NNMT-D197       gagccagaggcctttgactggtccccagtggtgacctatgtgtgtgatcttgaagggaac      360
WT-NNMT         gagccagaggcctttgactggtccccagtggtgacctatgtgtgtgatcttgaagggaac      360
                ************************************************************

NNMT-Y20        agagtcaagggtccagagaaggaggagaagttgagacaggcggtcaagcaggtgctgaag      420
NNMT-D197       agagtcaagggtccagagaaggaggagaagttgagacaggcggtcaagcaggtgctgaag      420
WT-NNMT         agagtcaagggtccagagaaggaggagaagttgagacaggcggtcaagcaggtgctgaag      420
                ************************************************************

NNMT-Y20        tgtgatgtgactcagagccagccactggggggccgtccccttaccccggctgactgcgtg      480
NNMT-D197       tgtgatgtgactcagagccagccactggggggccgtccccttaccccggctgactgcgtg      480
WT-NNMT         tgtgatgtgactcagagccagccactggggggccgtccccttaccccggctgactgcgtg      480
                ************************************************************

NNMT-Y20        ctcagcacactgtgtctggatgccgcctgcccagacctccccacctactgcagggcgctc      540
NNMT-D197       ctcagcacactgtgtctggatgccgcctgcccagacctccccacctactgcagggcgctc      540
WT-NNMT         ctcagcacactgtgtctggatgccgcctgcccagacctccccacctactgcagggcgctc      540
                ************************************************************

NNMT-Y20        aggaacctcggcagcctactgaagccagggggcttcctggtgatcatggatgcgctcaag      600
NNMT-D197       aggaacctcggcagcctactgaagccagggggcttcctggtgatcatggctgcgctcaag      600
WT-NNMT         aggaacctcggcagcctactgaagccagggggcttcctggtgatcatggatgcgctcaag      600
                ************************************************* ***********

NNMT-Y20        agcagctactacatgattggtgagcagaagttctccagcctcccccctgggccgggaggca      660
NNMT-D197       agcagctactacatgattggtgagcagaagttctccagcctcccccctgggccgggaggca      660
WT-NNMT         agcagctactacatgattggtgagcagaagttctccagcctcccccctgggccgggaggca      660
                ************************************************************

NNMT-Y20        gtagaggctgctgtgaaagaggctggctacacaatcgaatggtttgaggtgatctcgcaa      720
NNMT-D197       gtagaggctgctgtgaaagaggctggctacacaatcgaatggtttgaggtgatctcgcaa      720
WT-NNMT         gtagaggctgctgtgaaagaggctggctacacaatcgaatggtttgaggtgatctcgcaa      720
                ************************************************************

NNMT-Y20        agttattcttccaccatggccaacaacgaaggacttttctccctggtggcgaggaagctg      780
NNMT-D197       agttattcttccaccatggccaacaacgaaggacttttctccctggtggcgaggaagctg      780
WT-NNMT         agttattcttccaccatggccaacaacgaaggacttttctccctggtggcgaggaagctg      780
                ************************************************************

NNMT-Y20        agcagacccctgtga    795

NNMT-D197       agcagacccctgtga    795
WT-NNMT         agcagacccctgtga    795
                ***************
```

Fig. S1. Alignment of the site-specific mutation sequence of NNMT-D197 (blue) and NNMT-Y20 (purple) with NNMT-WT (NCBI Reference Sequence: NM_001372045.1).

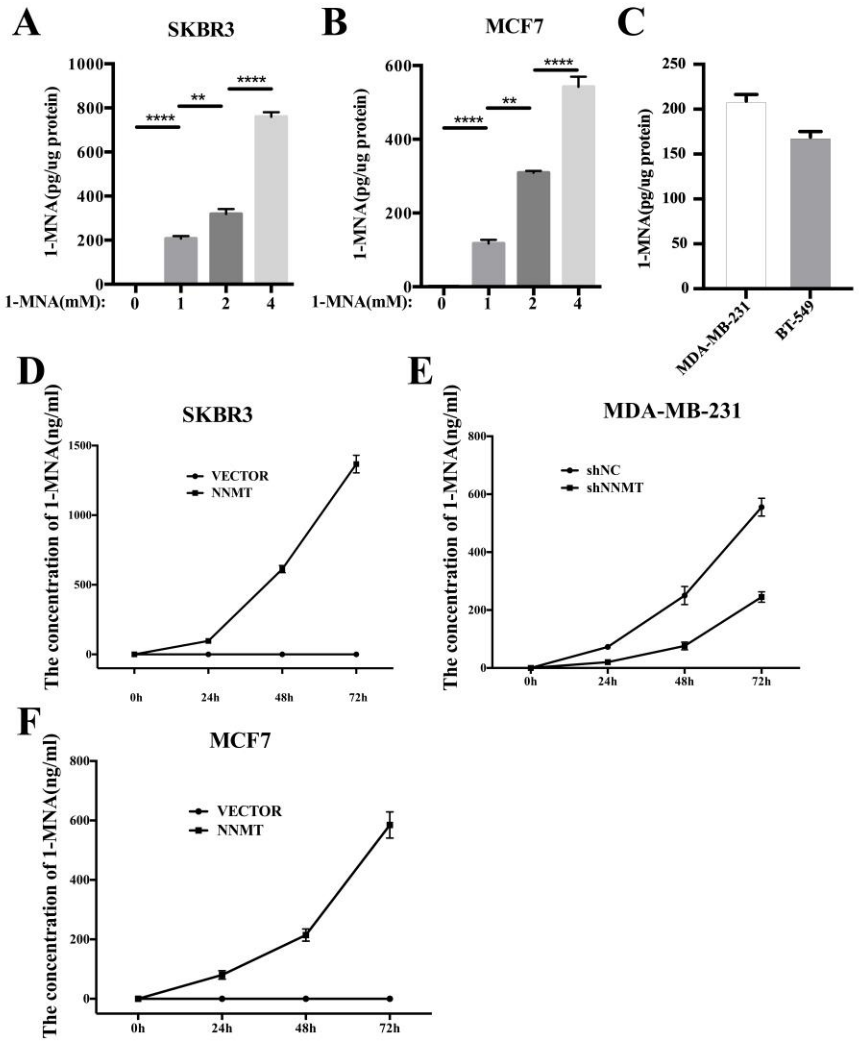

Fig. S2. LC-MS/MS detection of 1-MNA released by NNMT-expressing breast cancer, or 1-MNA intake by NNMT-free breast cancer cells. Amount of 1-MNA intake by SKBR3 cells **(A)** or MCF7 cells **(B)** was determined by LC-MS/MS, and standardized to total proteins concentration of cell lysates. C, LC-MS/MS detection of 1-MNA in the cell lysate of MDA-MB-231 cells and BT-549 cells, and amount of 1-MNA was standardized to total proteins concentration of cell lysates. **D-F,** quantification of 1-MNA level using LC-MS/MS in the supernatant of NNMT-overexpressing breast cancer cell lines (SKBR3/NNMT vs SKBR3/VECTOR, MCF7/NNMT vs MCF7/VECTOR) and NNMT-silencing breast cancer cell line (MDA-MB-231/1# vs MDA-MB-231/NC) at different culture time. Data are representative of three independent experiments. Data were presented as mean ± SEM. **, P<0.01; ***, P<0.001; ****, P<0.0001.

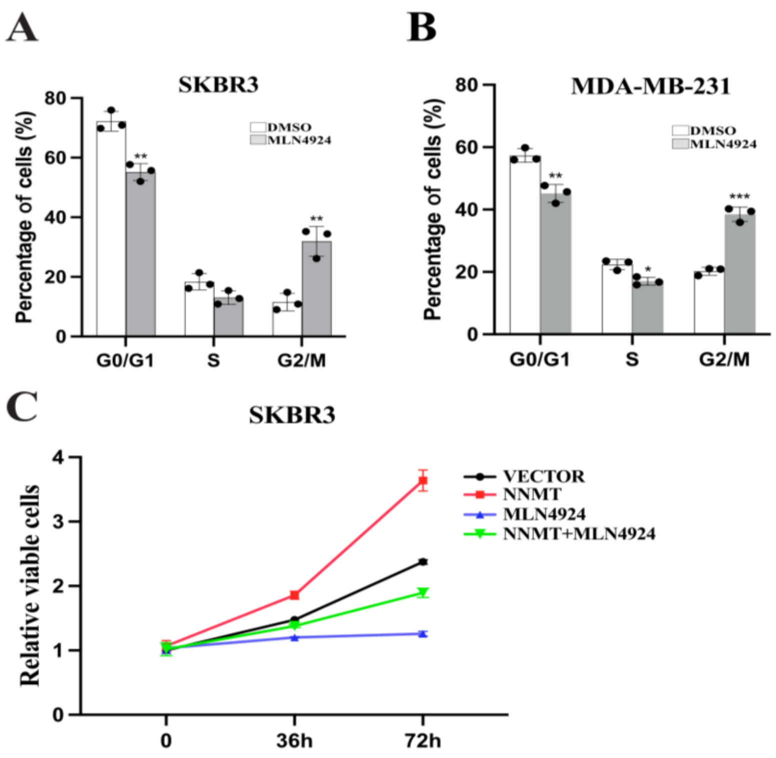

Fig. S3. A, flow cytometry analysis of cell-cycle progression of SKBR3 cells treated with DMSO or MLN4924(600nM, 24 h), and the results were calculated as percentage of cells in G0/G1 phase, S phase and G2/M phase. **B**, flow cytometry analysis of cell-cycle progression of MDA-MB-231 cells treated with DMSO or MLN4924(600nM, 24 h), and the results were calculated as percentage of cells in G0/G1 phase, S phase and G2/M phase. **C**, the relative cell viability was analyzed by Cell-Counting Kit-8 (CCK8) colorimetric assay in SKBR3/VECTOR cells, NNMT-over-expressing SKBR3 cells (SKBR3/NNMT), and SKBR3 cells(VECTOR or NNMT) treated with MLN4924 (600nM, 24 h). Data were presented as mean ± SEM. *, P<0.05; **, P<0.01; ***, P<0.001.

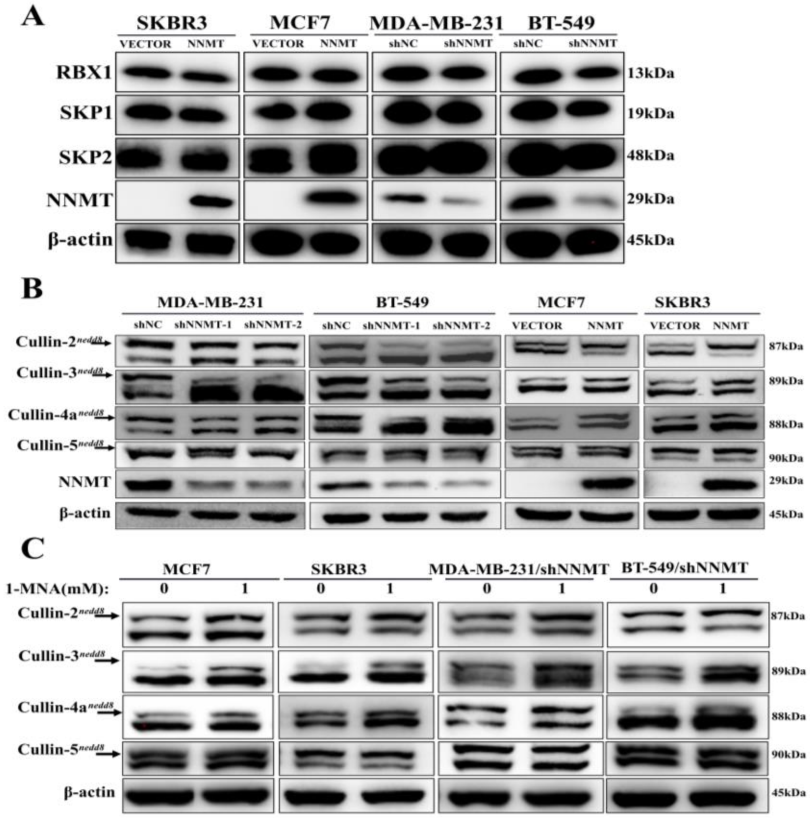

Fig. S4. A, western-blot detection of RBX1, SKP1, SKP2 and NNMT in NNMT over-expression cell line (MCF7/NNMT, SKBR3/NNMT) and NNMT specific shRNA (shNNMT) transfected cell line (BT-549 and MDA-MB-231), empty vector or control shRNA transfected cell lines were used as control. **B,** western-blot detection of cullin-1, cullin-2, cullin-3, cullin-4a and NNMT in NNMT over-expression cell line (MCF7/NNMT, SKBR3/NNMT) and two distinct NNMT specific shRNA (shNNMT-1 and shNNMT-2) transfected cell line (BT-549 and MDA-MB-231), empty vector or control shRNA transfected cell lines were used as control, and nedd8 conjugated cullins were indicated. **C**, MCF7, SKBR3, and NNMT silencing cells(MDA-MB-231/shNNMT and BT-549/shNNMT) were stimulated with or without 1-MNA(1.0mM) for 24hours, protein level of cullin-1, cullin-2, cullin-3 and cullin-4a was detected using western-blot, nedd8 conjugated cullins were indicated. Data are representative of three independent experiments.

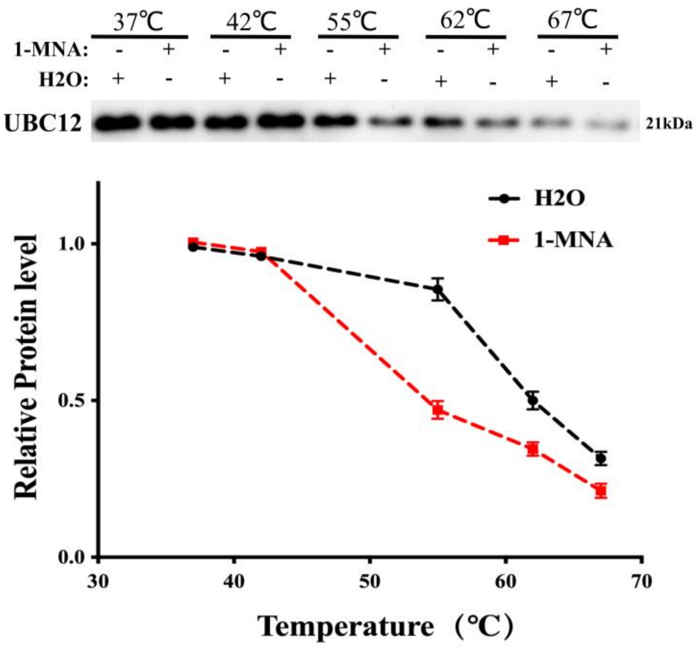

Fig. S5. Effect of 1-MNA on the thermo stability of UBC12. **C**ell lysate of MCF7 cells was divided into ten aliquots, and treated with 1-MNA(1mM) or H_2O (solvent), respectively. Then the samples were incubated at different temperatures as indicated (37°C, 42°C, 55°C, 62°C and 67°C) and protein level of UBC12 was detected using western-blot, the relative change of UBC12 proteins to starting temperature (37℃) was calculated.

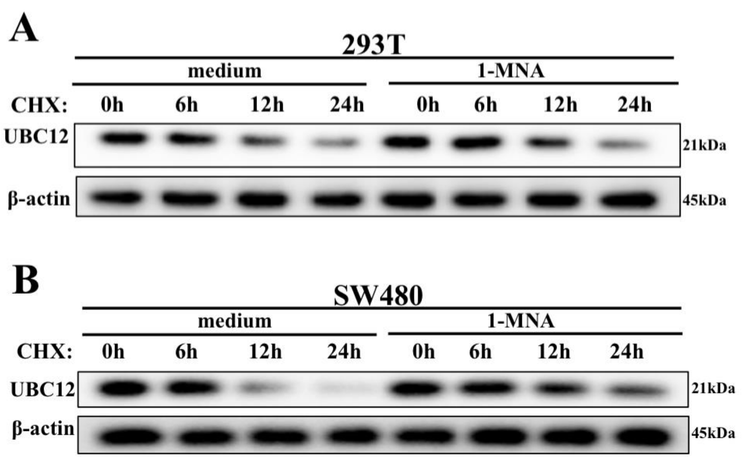

Fig. S6. 1-MNA delay the degradation process of UBC12 in 293T and SW480 cells. 293T cells **(A)** and SW480 cells **(B)** were treated with 1-MNA (1mM, 24 hrs) or DMSO, then immunoblot analysis of UBC12 proteins were done at indicated time points (0h, 6h, 12h, 24h) after CHX (30 ug/ml) treatment. Data are representative of three independent experiments.

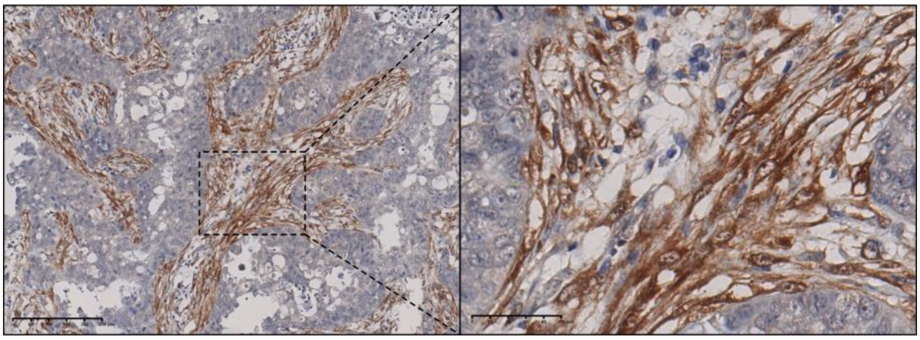

Fig. S7. NNMT expression in human breast cancer tissues was examined by IHC staining and the stroma was positively stained for NNMT.

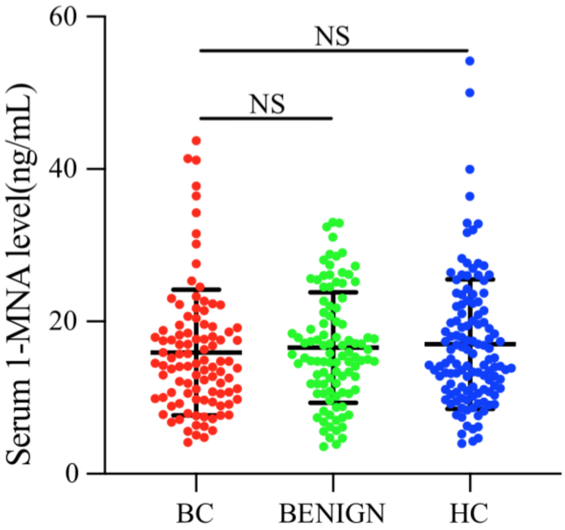

Supplementary Fig. S8. Serum concentrations of the 1-MNA in the BC patients, benign patients, and health controls. Datas are presented as mean±SD. Abbreviations: BC, breast cancer; BENIGN, breast benign disease; HC, health controls; NS, no significance.

Table S1. Construction frame of lentiviral vectors against NNMT.

**Lentiviral vecto**	**5’**	**STEM**	**Loop**	**STEM**	**3’**
NNMT shRNA 1#	T	GCTCAAGAGCAGCTACTACAT	CTCGAG	ATGTAGTAGCTGCTCTTGAGC	TTTTTTC
	TCGAGAAAAAA	GCTCAAGAGCAGCTACTACAT	CTCGAG	ATGTAGTAGCTGCTCTTGAGC	A
NNMT shRNA 2#	T	ACCCTCGGGATTACCTAGAAA	CTCGAG	TTTCTAGGTAATCCCGAGGGT	TTTTTTC
	TCGAGAAAAAA	ACCCTCGGGATTACCTAGAAA	CTCGAG	TTTCTAGGTAATCCCGAGGGT	A
shRNA NC	T	TTCTCCGAACGTGTCACGT	CTCGAG	ACGTGACACGTTCGGAGAA	TTTTTTC
	TCGAGAAAAAA	TTCTCCGAACGTGTCACGT	CTCGAG	ACGTGACACGTTCGGAGAA	A

Table S2. list of primers.

Name	Sequences(5’ to 3’)
NNMT Forward	GAGATCGTCGTCACTGACTACT
NNMT Reverse	CACACACATAGGTCACCACTG
UBC12 Forward	CCTGCCCAAGACGTGTGATA
UBC12 Reverse	CCCTGGCCCACCTTAAAACT
p27 Forward	TGCCCGAGTTCTACTACAGACC
p27 Reverse	CACCAAATGCGTGTCCTCAGA
β-actin Forward	CATGTACGTTGCTATCCAGGC
β-actin Reverse	CTCCTTAATGTCACGCACGAT

Table S3 is available at the following link:

https://doi.org/10.6084/m9.figshare.24785286.v1

Table S4 is available at the following link:

https://doi.org/10.6084/m9.figshare.24785289.v1
